# EV71 infection induces neurodegeneration via activating TLR7 signaling and IL-6 production

**DOI:** 10.1371/journal.ppat.1008142

**Published:** 2019-11-15

**Authors:** Zhen Luo, Rui Su, Wenbiao Wang, Yicong Liang, Xiaofeng Zeng, Muhammad Adnan Shereen, Nadia Bashir, Qi Zhang, Ling Zhao, Kailang Wu, Yingle Liu, Jianguo Wu

**Affiliations:** 1 Guangdong Key Laboratory of Virology, Institute of Medical Microbiology, Jinan University, Guangzhou, China; 2 State Key Laboratory of Virology, College of Life Sciences, Wuhan University, Wuhan, China; 3 School of Forensic Medicine, Kunming Medical University, Kunming, China; 4 State Key Laboratory of Agricultural Microbiology, College of Veterinary Medicine, Huazhong Agricultural University, Wuhan, China; Chang Gung University, TAIWAN

## Abstract

As a neurotropic virus, human Enterovirus 71 (EV71) infection causes hand-foot-and-mouth disease (HFMD) and may develop severe neurological disorders in infants. Toll-like receptor 7 (TLR7) acts as an innate immune receptor and is also a death receptor in the central nervous system (CNS). However, the mechanisms underlying the regulation of TLR7-mediated brain pathogenesis upon EV71 infection remain largely elusive. Here we reveal a novel mechanism by which EV71 infects astrocytes in the brain and induces neural pathogenesis *via* TLR7 and interleukin-6 (IL-6) in C57BL/6 mice and in human astroglioma U251 cells. Upon EV71 infection, wild-type (WT) mice displayed more significant body weight loss, higher clinical scores, and lower survival rates as compared with TLR7^-/-^ mice. In the cerebral cortex of EV71-infected mice, neurofilament integrity was disrupted, and inflammatory cell infiltration and neurodegeneration were induced in WT mice, whereas these were largely absent in TLR7^-/-^ mice. Similarly, IL-6 production, Caspase-3 cleavage, and cell apoptosis were significantly higher in EV71-infected WT mice as compared with TLR7^-/-^ mice. Moreover, EV71 preferentially infected and induced IL-6 in astrocytes of mice brain. In U251 cells, EV71-induced IL-6 production and cell apoptosis were suppressed by shRNA-mediated knockdown of TLR7 (shTLR7). Moreover, in the cerebral cortex of EV71-infected mice, the blockade of IL-6 with anti-IL-6 antibody (IL-6-Ab) restored the body weight loss, attenuated clinical scores, improved survival rates, reduced the disruption of neurofilament integrity, decreased cell apoptotic induction, and lowered levels of Caspase-3 cleavage. Similarly, in EV71-infected U251 cells, IL-6-Ab blocked EV71-induced IL-6 production and cell apoptosis in response to viral infection. Collectively, it’s exhibited TLR7 upregulation, IL-6 induction and astrocytic cell apoptosis in EV71-infected human brain. Taken together, we propose that EV71 infects astrocytes of the cerebral cortex in mice and human and triggers TLR7 signaling and IL-6 release, subsequently inducing neural pathogenesis in the brain.

## Author summary

Enterovirus 71 (EV71) infection causes aseptic meningitis, poliomyelitis-like paralysis and fatal encephalitis in infants. Besides an immune receptor, toll-like receptor 7 (TLR7) serves as a death receptor in central nervous system (CNS). However, the role of TLR7 in EV71-induced neural pathogenesis remains ambiguous. This study reveals a distinct mechanism by which EV71 induces neurodegeneration *via* TLR7 and interleukin-6 (IL-6). Upon EV71 infection, TLR7^-/-^ mice displayed less body weight loss, lower clinical score, and higher survival rate as compared with wild-type (WT) mice. Meanwhile, a severer histopathologic neurofilaments disruption, neurodegeneration and cell apoptosis were observed in brain of EV71-infected WT mice. IL-6 release, cell apoptosis, and Caspase-3 cleavage were attenuated by shRNA targeting TLR7 (shTLR7) in EV71-infected U251 cells. Moreover, anti-IL-6 antibody (IL-6-Ab) suppressed EV71-induced body weight loss, clinical score increase, and survival rate decrease as well as neurofilaments disruption and neurodegeneration in mice, and it also attenuated EV71-induced cell apoptosis and Caspase-3 cleavage in U251 cells. It’s retrospectively observed that TLR7 upregulation, IL-6 induction and astrocytic cell apoptosis in EV71-infected human brain. Therefore, TLR7 is required for neural pathogenesis by IL-6 induction upon EV71 infection.

## Introduction

Enterovirus 71 (EV71) is an RNA virus that causes hand-foot-mouth disease (HFMD), commonly causing mild symptom in infants, but in some cases also leading to severe diseases such as aseptic meningitis (AM), brain stem encephalitis (BSE), acute flaccid paralysis (AFP), and even fatal encephalitis in neonates [1–4]. As a neurotropic virus, the emerging EV71-related outbreaks have been the subject of great public health concern and are responsible for increased neurovirulence and mortality in the Asian-Pacific region [5, 6]. In HFMD epidemics in China from 2008 to 2012, more than 90% of deaths were associated with EV71 [7,8]. EV71 is thought to cause serious neurological disease with distinct clinicoradiological syndromes in affected patients, resulting in higher paresis-related morbidity in the gray matter of the brainstem or spinal cord [9]. However, the exact pathogenesis of EV71 infections, especially in cases where they cause severe neurological symptoms, remains poorly understood.

Toll-like receptor 7 (TLR7) is widely expressed in immune cells, intestinal cells, lung cells, and neural cells. As a pattern-recognition receptor (PRR), TLR7 can recognize viral RNA, oligomeric RNA, short interfering RNA (siRNA), and microRNA (miRNA) from pathogenic sources to activate intracellular signaling pathways and release appropriate cytokines [10–12]. Emerging evidence suggests that beyond its function as an immune receptor in immune cells, TLR7 also serves as a death receptor in various forms of non-infectious central nervous system (CNS) injury, such as ischemic stroke, Alzheimer’s disease, and morphine-mediated neurodegeneration [11, 13, 14].

In infectious contexts, TLR7 plays multiple roles relevant to neurological disorders and inflammatory responses in the CNS. When simian immunodeficiency virus (SIV) infects the brains of rhesus macaques, the secretion of microRNA21 activates the TLR7 pathway, leading to neurological disease [12]. Japanese encephalitis virus (JEV) infection elicits an elevation of inflammatory cytokine expression through TLR7 signaling to cause neuroinflammation [15]. Meningitis caused by Toscana virus (TOSV) is associated with a strong TLR7-mediated antiviral response in the CNS [16]. Upon EV71 infection, TLR7 usually acts as an inflammatory response modulator in intestinal epithelial cells and immune cells [17–20]. It remains unclear as to what role is played by TLR7 in the brain following EV71 infection, and the molecular mechanisms by which TLR7-mediates viral neural pathogenesis remain unclear.

In this study, we conducted experimental intracranial infections with EV71 in neonatal mice, and found a non-significant viral load in the cerebral cortex of both wild-type (WT) and TLR7 knockout (TLR7^-/-^) mice. Compared with EV71-infected WT mice, there was an obvious alleviation of neurological injury-related symptoms, histopathologic damage, and cell apoptosis, as well as lower brain IL-6 levels in EV71-infected TLR7^-/-^ mice. Further investigations revealed that intracranial IL-6 neutralization significantly relieved neurological injury-related symptoms and neural cell apoptosis in the brains of EV71-infected WT mice. Moreover, we demonstrated that EV71 preferentially infected and induced IL-6 production in murine brain astrocytes, and we further verified that TLR7 silencing and IL-6 blockade attenuated EV71-infected cell apoptosis *in vitro*. Finally, we retrospectively found TLR7 upregulation, IL-6 induction and astrocytic cell apoptosis in EV71-infected human brain. Thus, we identified a novel mechanism underlying TLR7-mediated neural pathogenesis involved in IL-6 induction in astrocytes of the brain upon EV71 infection.

## Results

### The evaluation of EV71-induced neuropathogenesis after intracranial injection in mice

**Fig 1:**
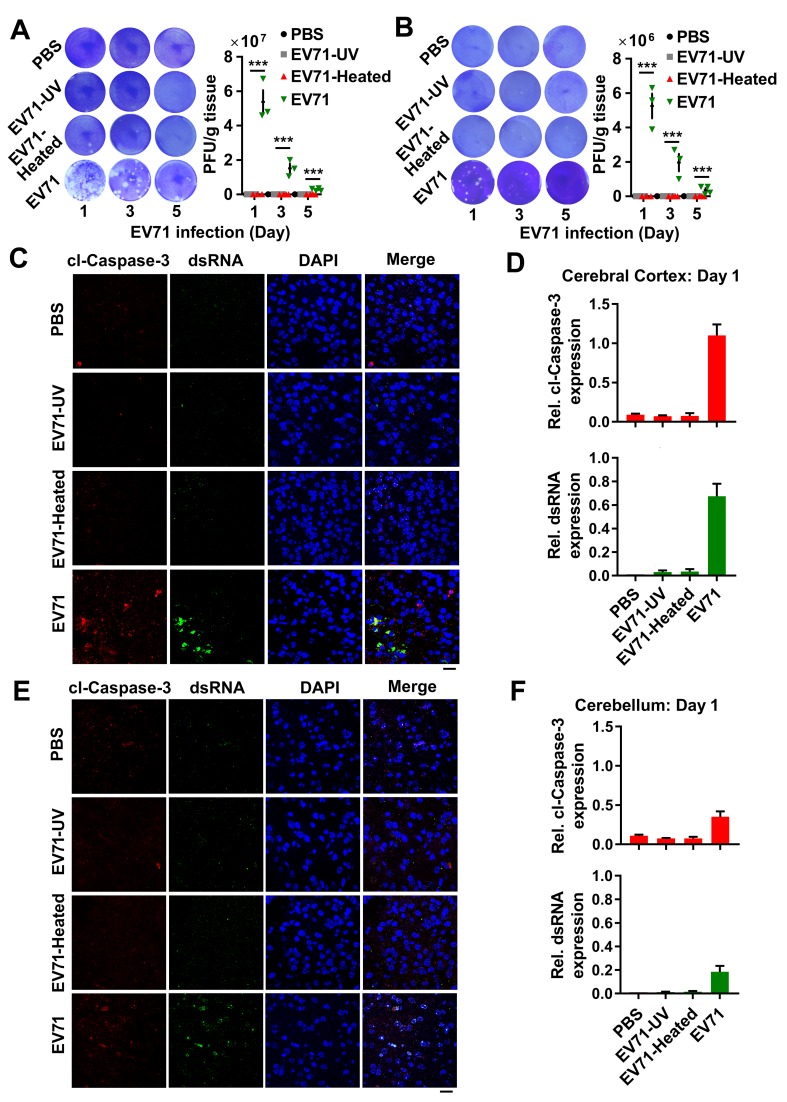
The neuropathogenesis assessment in intracranially EV71-infected mice. There-day-old WT mice were intracranially injected with 10 μl PBS, EV71-UV, EV71- Heated or EV71 per mouse (each group, n = 10–12) and sacrificed on day 1, 3 or 5 post-infection, respectively. (**A** and **B**) EV71 virus in cerebral cortex (A) or cerebellum (B) tissues homogenate was subjected to plaque formation assay. The EV71 titers in tissues of mice (per gram) were quantified. PFU, plaque formation unit. Data are shown as mean ± SD. ***, *P* < 0.001. (**C** and **D**) The cerebral cortex sections of mice on day 1 post-infection from different groups were fixed and subjected to immunostaining with cl-Caspase-3 (Red), dsRNA (Green), and DAPI (Blue) (C). The presentative images were acquired using fluorescence microscopy. Bar = 20 μm. The relative expression of cl-Caspase-3 and dsRNA was quantified using Image J software (D). Data are shown as mean ± SD. (**E** and **F**) The cerebellum sections of mice on day 1 post-infection from different groups were immunostained with cl-Caspase-3 (Red), dsRNA (Green), and DAPI (Blue) (E). The presentative images were acquired using fluorescence microscopy. Bar = 20 μm. The relative expression of cl-Caspase-3 and dsRNA was quantified using Image J software (F). Data are shown as mean ± SD.

Numerous animal models have been developed to study the pathogenesis upon EV71 infection, and the intracranial infection in suckling mice is established as an appropriate animal model to better understand EV71-associated neuropathogenesis [4]. To assess whether EV71 infection in mice brain caused neuropathogenesis, 3-day-old suckling WT mice were intracranially injected with PBS, UV-inactivated EV71 (EV71-UV), Heated-inactivated EV71 (EV71-Heated), and EV71. We found that EV71 robustly replicated on day 1 post-infection, and then declined from day 3 to 5 post-infection in the cerebral cortex tissues of EV71-infected mice, but not in mock-infected, UV-, or Heated-inactivated EV71-infected mice (Fig 1A and S1A Fig). We also observed a relatively low-level of EV71 replication in the cerebellum of EV71-infected mice (Fig 1B and S1B Fig).

The neuropathogenesis induced by neurotropic viruses including Zika virus (ZIKV) and Japanese encephalitis virus (JEV) is generally associated with neural cell apoptosis [21,22]. We further assessed the neural cell apoptosis induced by EV71 infection. On day 1 post-infection, the Caspase-3 cleavage (cl-Caspase-3) (activated apoptosis), and EV71 dsRNA were obviously detected in the cerebral cortex tissues of EV71-infected mice, but not in mock-infected, EV71-heated-infected, or EV71-UV-infected mice (Fig 1C and 1D). In addition, dsRNA had an obvious reduction from 3 days (S1C and S1D Fig) to 5 days (S1E and S1F Fig) post-infection, but cl-Caspase 3 became slightly higher from 3 days (S1C and S1D Fig) to 5 days (S1E and S1F Fig) post-infection. However, compared with the cerebral cortex of EV71-infected mice, we found a lower level of cl-Caspase-3 and dsRNA in the cerebellum of EV71-infected mice on day 1 post-infection (Fig 1E and 1F) and similar observations were obtained on day 3 (S2A and S2B Fig) and day 5 (S2C and S2D Fig) post-infection. Thus, these results reveal that EV71 robustly replicates and induces cell apoptosis in the cerebral cortex of EV71-infected mice, but exhibits relatively low levels of viral replication and cell apoptosis in the cerebellum of EV71-infected mice in the intracranial injection model.

### *TLR7* is deleted in the brain tissues of *TLR7*^-/-^ mice

**Fig 2:**
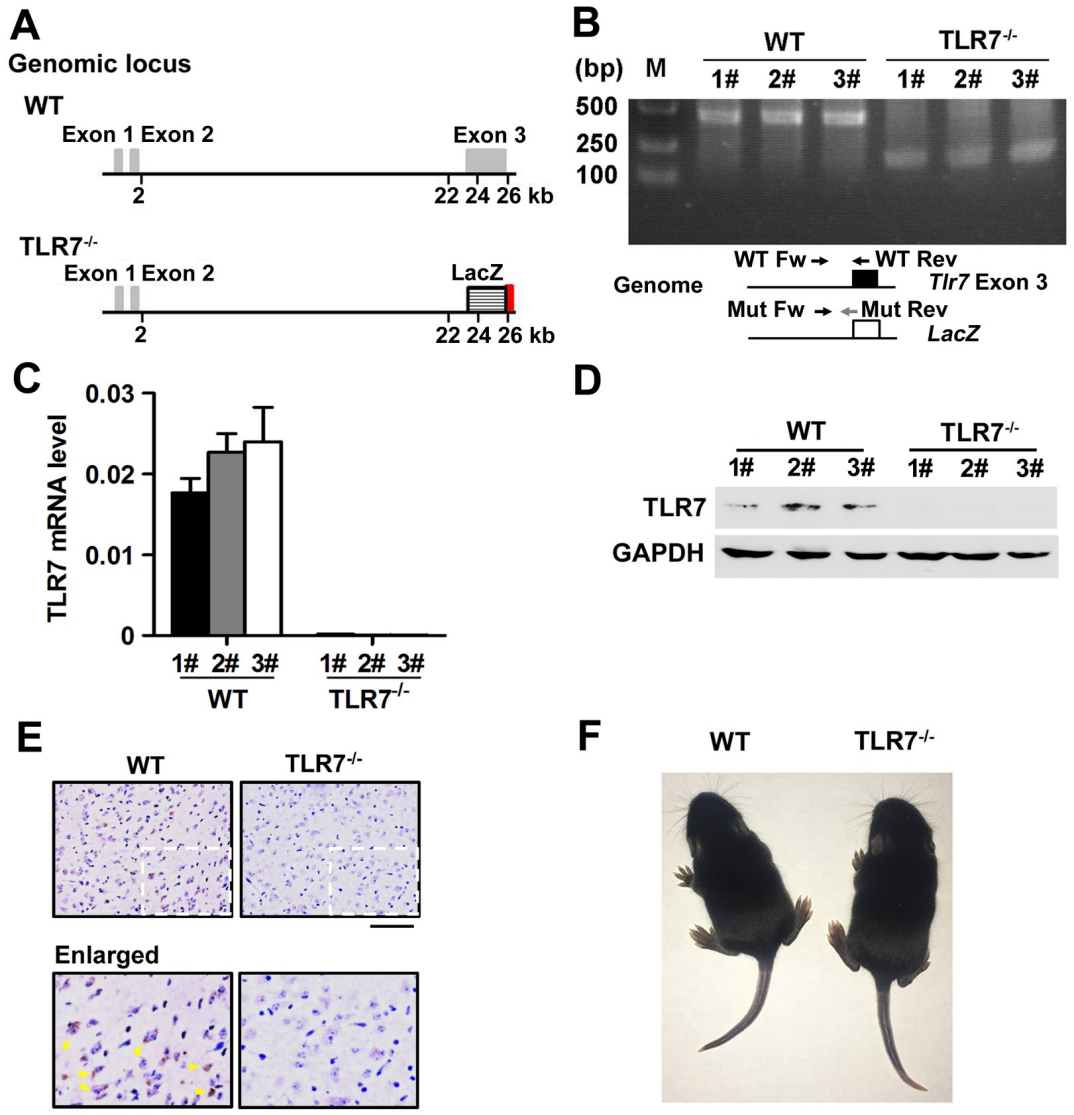
*TLR7* is deleted in the brain tissues of *TLR7*^-/-^ mice. (**A**) The map of genomic locus of *TLR7* gene in wild-type (WT) and TLR7 deficiency (TLR7^-/-^). In the generation of *TLR7* gene knockout mice, the *lacZ* reporter with a stop code was introduced under the gene's promoter in the replace of Exon 3 of *TLR7* gene. A segment of exon 3 was replaced by a *LacZ* gene and *LoxP*-flanked neomycin resistance cassette. The targeting vector was introduced to embryonic stem (ES) cells. (**B**–**E**) The 3-day-old mice of WT and TLR7^-/-^ homozygotes (each group, n = 3) were sacrificed and the mice brain tissue were collected. The genome DNAs were extracted from individual mice brain tissue and then *TLR7* gene deletion in genome was identified by PCR with specific primers as followed (B). WT Forward (WT Fw): 5’-AGGGTATGCCGCCAAATCTAAAG-3’; WT Reverse (WT Rev): 5’-ACCTTTGTGTGCTCCTGGAC-3’ and Mutant (TLR7 deletion) (Mut Rev): 5’-TCATTCTCAGTATTGTTTTGCC-3’. The lengths of DNA products are 183 bp, 183 bp and 454 bp, 454 bp for Mutant, Heterozygote, and WT, respectively. The mRNAs were extracted from individual mice brain tissues and then the *TLR7* mRNA level was measured by qPCR with specific primers. The relative *TLR7* expression value was determined by the ratio of TLR7/GAPDH (C). The proteins were extracted from individual mice brain tissues and then detected by Western blotting with targeted antibodies (D). The cerebral cortex sections of mice brain were fixed and subjected to immunohistochemical (IHC) staining with TLR7 antibody. The positive TLR7 staining (Brown) was indicated by arrows in yellow (E). The presentative images were acquired using light microscopy. Bar = 100 μm. (**F**) Photographs of WT neonatal mouse and TLR7^-/-^ neonatal mouse.

Our previous work has identified that TLR7 orchestrates the inflammatory and innate immune responses upon EV71 infection in macrophages [19]. This study further sought to determine whether TLR7 plays a role in EV71-infected neuropathogenesis *in vivo* utilizing a TLR7 knock-out (TLR7^-/-^) mouse model. Initially, the genetic characteristics of the *TLR7* gene in brain tissues were determined. By assessing the *TLR7* gene (Fig 2A), we systematically determined that *TLR7* DNA sequences (Fig 2B), *TLR7* mRNA expression (Fig 2C), and TLR7 protein production (Fig 2D) were present in the brain tissues of WT mice, whereas they were not detected in the brains of TLR7^-/-^ mice. Similarly, immunohistochemistry (IHC) staining indicated that TLR7 protein was present in the cerebral cortex of WT mice, whereas it was not produced in the cerebral cortex of TLR7^-/-^ mice (Fig 2E). Thus, these results confirm that the *TLR7* gene was successfully deleted in the brain tissues of homozygous TLR7^-/-^ mice. Of note, there were no visible differences in behavior and growth between WT neonatal mice and TLR7^-/-^ neonatal mice (Fig 2F).

### TLR7 promotes EV71-induced neural pathogenesis in the murine cerebral cortex

**Fig 3:**
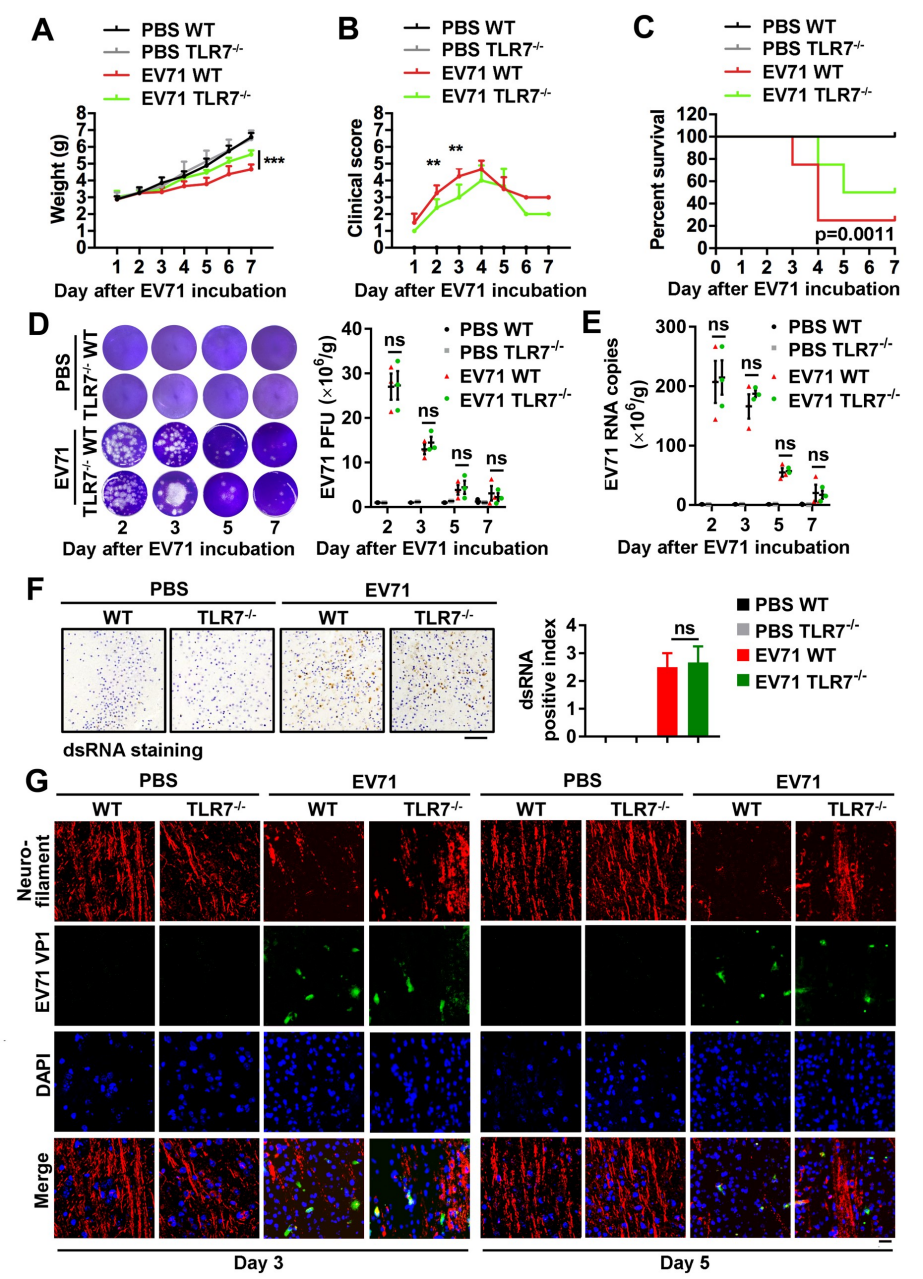
TLR7 promotes EV71-induced neural pathogenesis in the murine cerebral cortex. (**A**–**C**) Three-day-old neonatal WT and TLR7^-/-^ mice were intracranially injected with 10 μl PBS or EV71 per mouse (each PBS group, n = 8; each EV71 group, n = 10). The weight (A), clinical score (B), and Kaplan-Meier survival curve (C) were recorded from day 1 to 7 post EV71 incubation. Data are shown as mean ± SD. **, *P* < 0.01; ***, *P* < 0.001. (**D**–**G**) WT mice and TLR7^-/-^ mice mock-infected or EV71-infected were sacrificed on 2, 3, 5, and 7 days post-infection (each group, n = 3–5). EV71 virus from brain tissues homogenate was subjected to plaque formation assay. The EV71 titers in brain tissues of mice (per gram) were quantified (D). Graphs show means ± SD. ns, non-significant. The RNA was extracted from the brain tissues and EV71 RNA copies were quantified in brain tissues of mice (per gram) using absolute quantitative PCR (E). Graphs show means ± SD. ns, non-significant. The mice brain sections on day 3 post EV71 incubation were fixed and subjected to IHC staining with dsRNA antibody (Brown) (F). The presentative images were acquired using light microscopy. Bar = 100 μm. The dsRNA relative expression was shown as a dsRNA positive index and quantified with Image J software. Graphs show means ± SD. ns, non-significant. Immunostaining of the brain’s cortex from day 3 and 5 post EV71 incubation was probed with Neurofilament (Red), EV71 VP1 (Green), and stained with DAPI (Blue) (G). The presentative images were acquired using fluorescence microscopy. Bar = 20 μm.

The effects of TLR7 on EV71 infection-induced neuropathogenesis were then evaluated in WT and TLR7^-/-^ mice. Three-day-old neonatal mice were intracranially injected with or without EV71, in accordance with a previously established intracranial infection model for assessing EV71 neurovirulence [4]. As compared with EV71-infected TLR7^-/-^ mice, infected WT mice exhibited significantly greater loss of body weight (Fig 3A), higher clinical scores (consistent with hind-limb paralysis) (Fig 3B), and lower survival rates (Fig 3C), suggesting that TLR7 is involved in promoting EV71-induced neurovirulence in mice.

Next, the role of TLR7 in regulating EV71 replication in the murine brains was verified. Notably, there was no significant difference in EV71 load in the brain tissues of WT and TLR7^-/-^ mice (Fig 3D and 3E). We found that the PFU value of EV71 was robustly high from day 2 to 3 post-infection, and then declined from day 5 to 7 post-infection in the brain tissues of both infected WT and TLR7^-/-^ mice (Fig 3D). Similar results were observed that the no significant difference in EV71 RNA levels in the brain tissues of WT and TLR7^-/-^ mice (Fig 3E). Parallel IHC staining for the EV71 VP1 protein revealed that VP1 was produced in the cerebral cortex, and that there was no significant difference in VP1 levels between WT and TLR7^-/-^ mice (S3A Fig), whereas the viral protein was a relatively low-level expressed in the cerebellum (S3B Fig). Additional IHC staining with an antibody against EV71 dsRNA demonstrated that EV71 dsRNA was present with no significant difference in the cerebral cortex of both WT and TLR7^-/-^ mice (Fig 3F). These results suggest that TLR7 does not play an antiviral role in the brain upon EV71 infection.

To further explore the role of TLR7 in neuropathogenesis induced upon EV71 infection, we analyzed the expression status of neurofilament protein. These 10-nm intermediate filaments of neurons provide structure and mechanoresistance in the brain, and disruption of neurofilament organization and expression is a characteristic of certain neurological disorders [11, 23]. Here, we examined the effect of TLR7 on neurofilament organization and expression upon EV71 infection via immunohistochemical analysis of the cerebral cortex on Day 3 and 5 after EV71 infection in WT and TLR7^-/-^ mice with neurofilament staining. Fluorescence microscopy showed that in PBS-treated mice, neurofilament integrity was intact in the cortexes of both WT and TLR7^-/-^ mice, whereas upon EV71 infection, the integrity of neurofilaments was disrupted and the levels of neurofilaments were reduced with the progressive viral infection in the cortex of EV71-infected WT mice relative to TLR7^-/-^ mice (Fig 3G), indicating that TLR7 plays a critical role in facilitating EV71-induced neurodegeneration and neuropathogenesis. Taken together, our results illustrate that TLR7 facilitates EV71-induced neuropathogenesis in the cerebral cortex in mice.

### TLR7 is required for EV71-induced neural cell apoptosis in the brain

**Fig 4:**
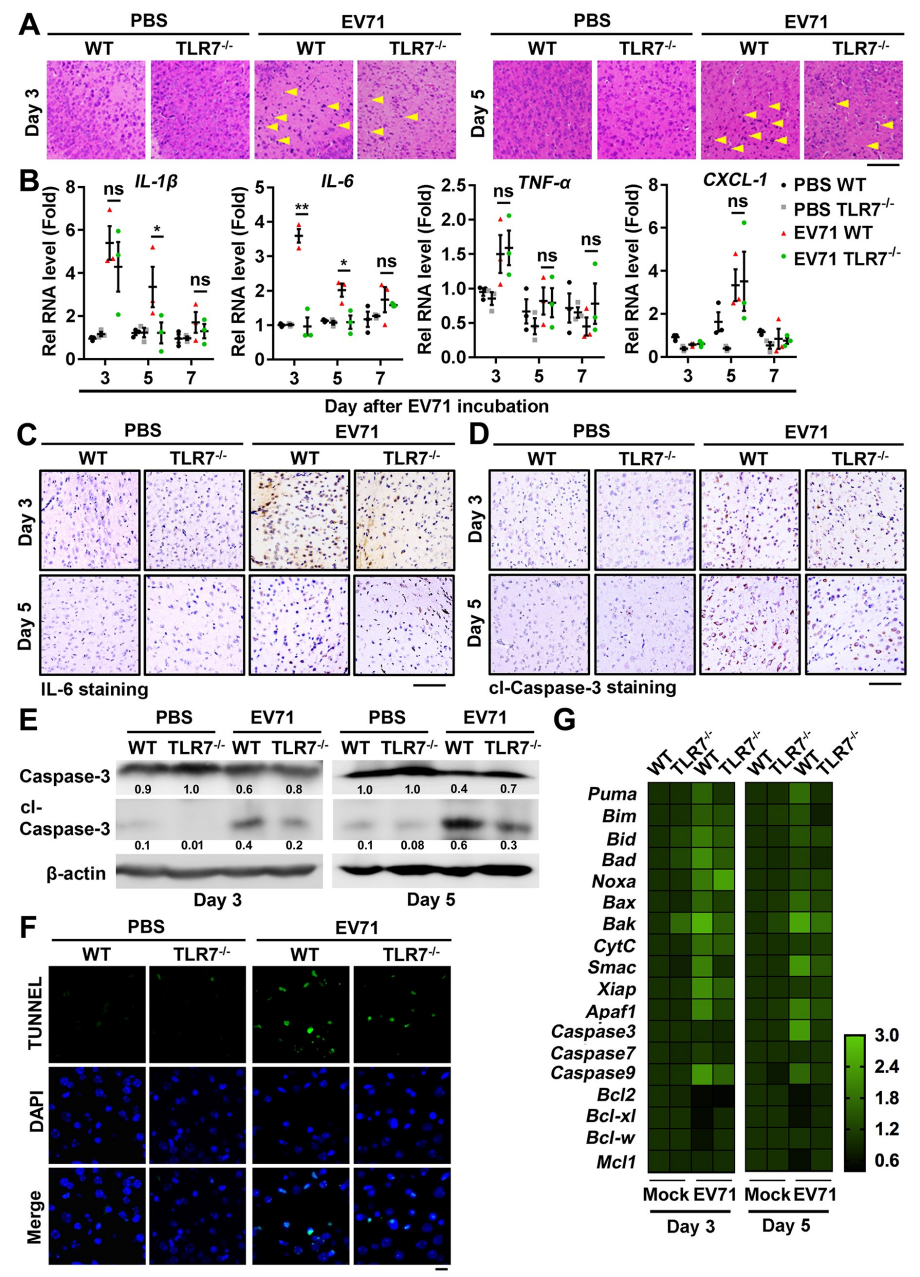
TLR7 is required for EV71-induced neural cell apoptosis in the brain. (**A**) The mice cerebral cortex sections from WT and TLR7^-/-^ mice mock-infected or EV71-infected for 3 or 5 days were subjected to hematoxylin-eosin (H&E) staining. The typical inflammatory cells infiltration or neurodegeneration were indicated by yellow arrows. The presentative images were acquired using light microscopy. Bar = 100 μm. (**B**) The WT and TLR7^-/-^ mice were sacrificed on 3, 5, and 7 days post-infection (each group, n = 3). The RNA was extracted from the brain tissues and the levels of *IL-1β*, *IL-6*, *TNF-α*, and *CXCL-1* mRNAs were measured by qPCR. Data are shown as mean ± SD. **, *P* < 0.01; ***, *P* < 0.001; ns, non-significant. (**C** and **D**) The WT and TLR7^-/-^ mice brain sections on 3 and 5 days post-infection were subjected to IHC staining with anti-IL-6 antibody (Brown) (C) and anti-cleaved-Caspase-3 (cl-Caspase-3) antibody (Brown) (D). The presentative images were acquired using light microscopy. Bar = 100 μm. (**E**) The proteins were extracted from individual mice brain tissues and then detected by Western blotting with targeted antibodies. Relative Caspase-3 or cl-Caspase-3 protein expression to internal control is quantified using Image J software. (**F**) The brain sections from mice on day 3 post EV71 incubation were subjected to immunostained with TUNNEL (Green) and DAPI (Blue). The presentative images were acquired using fluorescence microscopy. Bar = 20 μm. (**G**) The RNA was extracted from the brain tissues on day 3 and 5 post EV71 incubation. The levels of apoptosis-associated genes mRNAs were measured by qPCR. Data are shown as fold changes of RNA expression compared to mock samples (PBS WT group).

Considering the neuropathogenesis induced by EV71 is generally associated with neural cell apoptosis, we further assessed the role of TLR7 in regulating neural cell apoptosis induced by EV71 infection. WT mice and TLR7^-/-^ mice were treated with PBS or infected with EV71 for 3 and 5 days, and then rates of cellular apoptosis and neuropathogenesis were assessed in cortex samples. Histopathological assessments revealed that typical inflammatory cell infiltration and neurodegeneration were evident in the cerebral cortex of infected WT mice, but were largely absent in the cerebral cortex of infected TLR7^-/-^ mice (Fig 4A), indicating that TLR7 plays a role in neural injury and neurodegeneration upon EV71 infection.

It has been reported that the neuroinflammatory response is an important indicator of neural injury [24]. We next sought to assess the role of TLR7 in regulating expression of pro-inflammatory cytokines, including interleukin-1β (IL-1β), interleukin-6 (IL-6), tumor necrosis factor-α (TNF-α), and C-X-C motif chemokine 1 (Cxcl-1) upon EV71 infection. Our results revealed that *IL-1β* mRNA level was higher in the infected cerebral cortex of WT mice on 3 days post-infection as compared with infected TLR7^-/-^ mice (Fig 4B). Notably, *IL-6* mRNA was induced in the cerebral cortex of infected WT mice on 3 and 5 day post-infection, whereas it only slightly induced in the cortex of infected TLR7^-/-^ mice (Fig 4A). In addition, there were no differences in the levels of *TNF-α* or *CXCL-1* mRNA between EV71-infected WT and TLR7^-/-^ cerebral cortex samples (Fig 4B). Moreover, IHC staining using an antibody against endogenous IL-6 revealed that IL-6 protein production was induced in the cerebral cortex of infected WT mice, whereas it was only slightly induced in the cerebral cortex of infected TLR7^-/-^ mice (Fig 4C). Taken together, these results suggest that IL-6 is induced by EV71 and plays a primary role in the neuropathogenesis in mice induced upon EV71 infection.

Additionally, the role of TLR7 in regulating Caspase-3 cleavage was assessed. IHC staining revealed that cleaved-Caspase-3 (cl-Caspase-3) was barely detected in the cerebral cortex of both mock-infected WT and TLR7^-/-^ mice (Fig 4D, left); whereas upon EV71 infection, cl-Caspase-3 protein was induced and the level of cl-Caspase-3 was much higher in cerebral cortex of infected WT mice as compared with that in infected TLR7^-/-^ mice (Fig 4D, right). Similarly, Western blotting further confirmed that cl-Caspase-3 was largely absent in cerebral cortex of both mock-infected WT and TLR7^-/-^ mice, whereas upon EV71 infection, cl-Caspase-3 protein was induced and the levels of cl-Caspase-3 were enhanced in infected WT mice relative to infected TLR7^-/-^ mice (Fig 4E). These data suggest that TLR7 is required for cell apoptosis in the cerebral cortex of mice upon EV71 infection. Furthermore, TUNNEL staining indicated that cell apoptosis was largely absent in brain sections of mock-infected WT mice and TLR7^-/-^ mice (Fig 4F, left); however, upon EV71 infection, cell apoptosis was apparent in brain sections of both WT and TLR7^-/-^ mice, with the level of apoptosis being higher in infected WT mouse brain sections relative to infected TLR7^-/-^ mice brain sections (Fig 4F, right), further suggesting that TLR7 is involved in mediating apoptotic cell death in the brain upon EV71 infection.

Given that in apoptosis-related pathways, specific sets of genes function as apoptotic initiators, apoptosis effectors, and anti-apoptotic factors in senescent or damaged cells [25], we next thought to establish the role of TLR7 in regulating the expression of 18 apoptosis-related genes in the brain tissues of WT and TLR7^-/-^ mice with or without EV71 infection (Table 1). Our results revealed that the apoptotic initiators p53 upregulated modulator of apoptosis (Puma), BCL-2 interacting mediator of cell death (Bim, also known as Bcl2L11), BH3 interacting domain death agonist (Bid), and BCL2-associated agonist of cell death (Bad) were up-regulated in EV71-infected WT mouse brain tissues, whereas the anti-apoptotic factors B-cell lymphoma-2 (Bcl-2), B-cell lymphoma-extra large (Bcl-XL), and BCL-2 like 2 (Bcl-W) were down-regulated in these same samples (Fig 4G). We also found that the altered regulation of these genes was markedly attenuated in infected TLR7^-/-^ mice brain tissues relative to those from WT mice (Fig 4F). These results suggest that TLR7 facilitates the activation of apoptotic initiators and the repression of anti-apoptotic factors. Taken together, our results reveal that TLR7 is required for neural cell apoptosis in the murine brain upon EV71 infection.

**Table 1:** List of apoptosis-associated genes information in this study.

Gene Name	Description	Category
PUMA	p53 upregulated modulator of apoptosis, BCL2 binding component 3	Pro-apoptotic initiators
BIM	BCL-2 interacting mediator of cell death, BCL2 like 11	
BID	BH3 interacting domain death agonist	
BAD	BCL2 associated agonist of cell death	BCL2/MCL1 inhibitors
NOXA	Phorbol-12-myristate-13-acetate-induced protein 1	
BAX	BCL2 associated X, apoptosis regulator	Apoptosis effectors
BAK	BCL2 antagonist/killer 1	
CytC	Cytochrome c, somatic	
SMAC	Diablo IAP-binding mitochondrial protein	
XIAP	X-linked inhibitor of apoptosis	
APAF1	Death-associated APAF1-related killer	
BCL-2	B-cell lymphoma-2, apoptosis regulator	Anti-apoptotic factors
BCL-XL	B-cell lymphoma-extra large, BCL-2 like 1	
BCL-W	B-cell lymphoma-W, BCL-2 like 2	
MCL1	MCL1 apoptosis regulator, BCL2 family member	
Caspase-3	Cysteine-aspartic acid protease-3	Caspase cascade
Caspase-7	Cysteine-aspartic acid protease-7	
Caspase-9	Cysteine-aspartic acid protease-9	

### IL-6 plays a crucial role in TLR7-mediated neural pathogenesis upon EV71 infection

**Fig 5:**
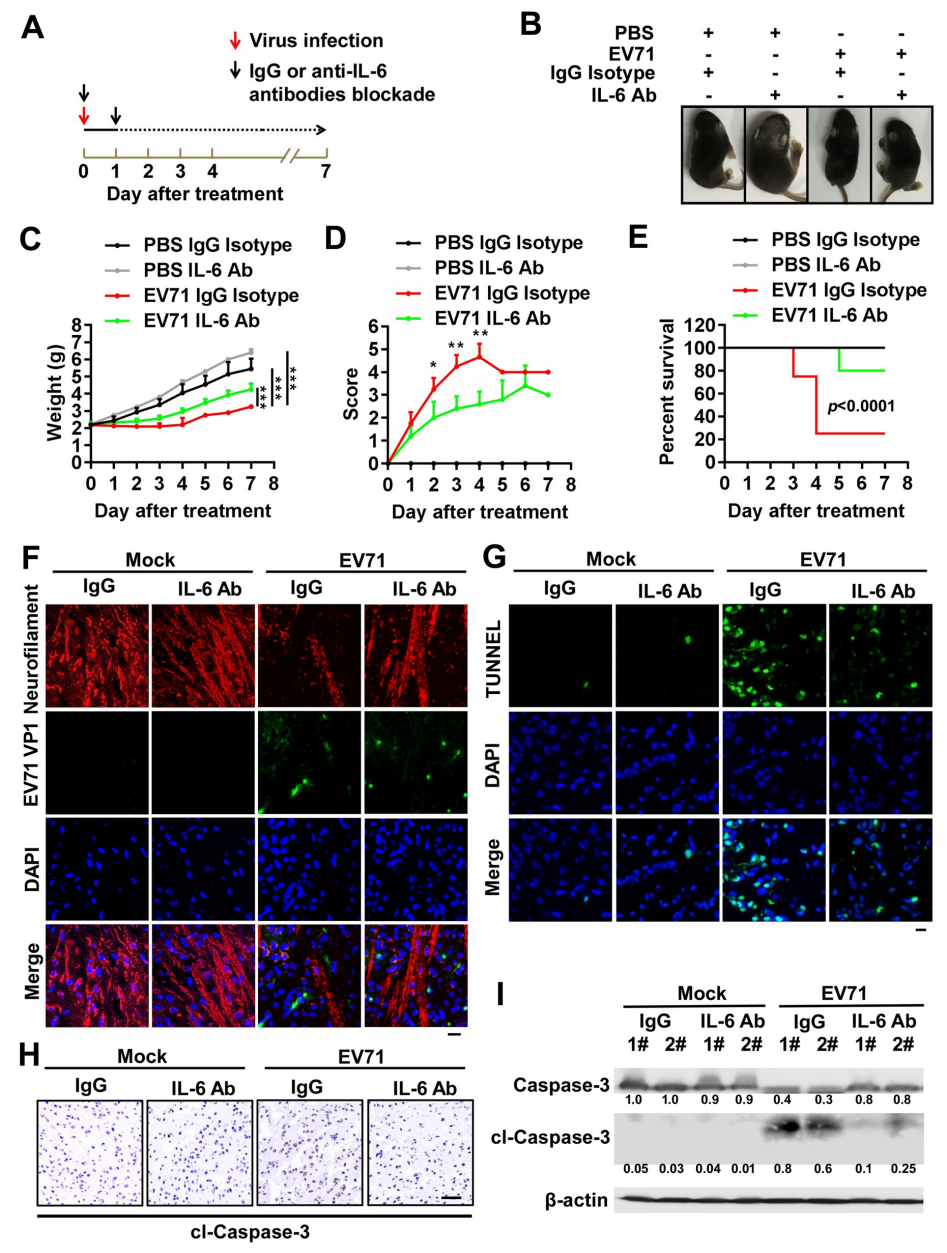
IL-6 plays a crucial role in TLR7-mediated neural pathogenesis upon EV71 infection. (**A**) The schedule of IgG isotype and anti-IL-6 antibodies blockade treatment after EV71 incubation in 3-day-old WT mice brain. Neonatal WT mice were intracranially injected with 10 μl PBS or EV71 per mouse (each group, n = 8), and separately intracranially treated with IgG isotype or anti-IL-6 antibody (8 μg/per mouse) twice at Day 0 and 1 (black arrows). (**B**) The presentative images of mice on day 5 in indicated groups. (**C**–**E**) The weight (C), clinical score (D), and Kaplan-Meier survival curve (E) were recorded from 1 to 7 days post-infection. Data are shown as mean ± SD. **, *P* < 0.01; ***, *P* < 0.001. (**F** and **G**) The brain sections of mice on day 5 in different groups were subjected to immunostained with DAPI (Blue), Neurofilament (Red) and EV71 VP1 (Green) (F) or TUNNEL (Green) (G). The presentative images were acquired using fluorescence microscopy. Bar = 20 μm. (**H**) The mice brain sections from day 5 in different groups were subjected to IHC staining with cl-Caspase-3 antibody (Brown). The presentative images were acquired using light microscopy. Bar = 100 μm. (**I**) The proteins were extracted from individual mice brain tissues on day 5 in different groups and then detected by Western blotting with targeted antibodies. Relative Caspase-3 or cl-Caspase-3 protein expression to internal control is quantified using Image J software.

In immune cells, EV71 activates TLR7 signaling to induce the production of multiple inflammatory cytokines, including IL-6 [19]. Elevated levels of hippocampal IL-6 are thought to be related to neurodegeneration in the early stages of Alzheimer’s disease [26]. As TLR7 mediated IL-6 expression and cell apoptosis in the cerebral cortex of EV71-infected mice, we further assessed the role of IL-6 in TLR7-mediated neuropathogenesis upon EV71 infection. Neonatal WT mice were intracranially injected with EV71 and then treated with isotype IgG (IgG) or anti-IL-6 antibody (IL-6-Ab) (Fig 5A). Remarkably, upon EV71 infection, the body size of IgG-treated mice was significantly reduced, whereas the body size of IL-6-Ab-treated mice was only slightly reduced relative to uninfected controls (Fig 5B). We further found that upon EV71 infection, IgG-treated mice displayed significantly lower body weight (Fig 5C), exhibited much higher clinical scores (Fig 5D), and had lower survival rates (Fig 5E), than did IL-6-Ab-treated mice (Fig 5C–5E).

Due to some specific secreted proteins and peptides entering to the cerebrospinal fluid (CSF) and blood circulation [27], the IL-6 protein level and EV71 viral load in CNS and peripheral tissues after IL-6 antibody neutralizing administration was assessed. As expected, IL-6 protein levels were significantly reduced by IL-6-Ab neutralization in the cerebral cortex (S4A Fig), cerebellum tissues (S4B Fig), as well as CSF (S4C Fig) of both mock-infected or EV71-infected mice. However, IL-6 protein level didn’t change in peripheral blood (S4D Fig) of both mock-infected or EV71-infected mice after IL-6-Ab neutralization. Moreover, there was no significant difference in virus titers in the cerebral cortex (S4E Fig), cerebellum tissues (S4F Fig), as well as CSF (S4G Fig) of IgG-treated and IL-6-Ab-treated EV71-infected mice. Notably, EV71 virus was not detected in the peripheral blood of EV71-infected mice (S4H Fig). The similar results were also observed in the tissues of cerebral cortex (S5A and S5B Fig) and cerebellum tissues (S5C and S5D Fig) of different groups of mice. In addition, there was a low level of IL-6 protein and EV71 load in spinal cord (S6A and S6B Fig), while their expressions were barely detected in skeletal muscle (S6C and S6D Fig) of EV71-infected mice. These results revealed that EV71-induced IL-6 induction could be reduced, whereas EV71 viral load was not affected after IL-6-Ab neutralization in mice brain, suggesting that IL-6 blockade mediated protection is independent of the virus load.

Moreover, immunohistochemical staining for neurofilaments revealed that in mock-infected mice, the integrity of neurofilaments was intact in the cerebral cortex of both IgG-treated and IL-6-Ab-treated mice, whereas upon EV71 infection, the integrity of neurofilaments was disrupted and neurofilament levels were reduced with the progressive viral replication in the cerebral cortex of IgG-treated mice relative to those treated with IL-6-Ab (Fig 5F). These results suggest that IL-6 plays a role in promoting EV71-induced neurodegeneration and neuropathogenesis in mice.

Next, the effects of IL-6 on EV71-induced cell apoptosis were assessed. TUNNEL staining revealed that in mock-infected mice, cell apoptosis was barely detectable in the cerebral cortex of IgG-treated and IL-6-Ab treated mice (Fig 5G, left). However, upon EV71 infection, cell apoptosis occurred in the cerebral cortex of both IgG-treated and IL-6-Ab treated mice, while the level of cell apoptosis was higher in the cerebral cortex of IgG-treated mice as compared with IL-6-Ab treated mice (Fig 5G, right), suggesting that IL-6 promotes EV71-induced cell apoptosis in the cerebral cortex. Parallel IHC staining revealed that cl-Caspase-3 levels were largely undetectable in brain sections of both IgG-treated and IL-6-Ab treated mice (Fig 5H, left), whereas upon EV71 infection, cl-Caspase-3 was induced and the level of cl-Caspase-3 was higher in the brain sections of IgG-treated mice as compared with those in IL-6-Ab treated mice (Fig 5H, right). Consistent with these findings, Western blotting revealed a lack of cl-Caspase-3 in brain samples from mock-infected IgG- and IL-6-Ab treated mice, whereas upon EV71 infection, cl-Caspase-3 was induced in the brain sections of both IgG-treated and IL-6-Ab treated mice, and the levels of cl-Caspase-3 were higher in the brain sections of IgG-treated mice as compared with those in IL-6-Ab treated mice (Fig 5I). Thus, these data revealed that IL-6 plays a dominant role in TLR7-mediated neuropathogenesis upon EV71 infection *in vivo*.

### EV71 preferentially infects astrocytes in the mouse brain and induces IL-6 production *via* TLR7

**Fig 6:**
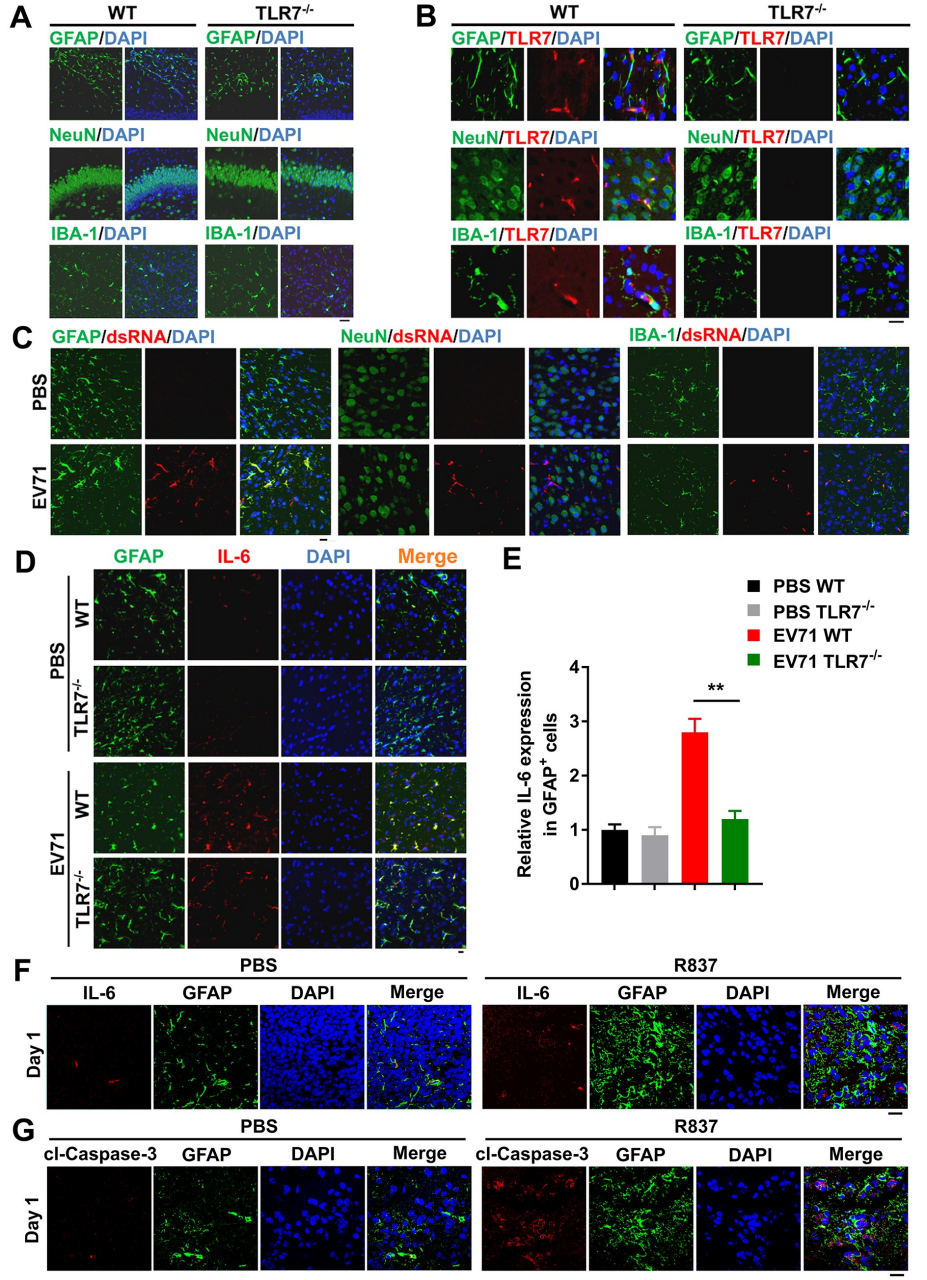
EV71 preferentially infects astrocytes of mice brain and induces IL-6 production *via* TLR7. (**A** and **B**) The neonatal (6-day-old) WT and TLR7^-/-^ mice were sacrificed and the sections of mice cerebral cortex or hippocampus were subjected to immunofluorescence (IF) staining with DAPI (Blue) and neural cells markers (GFAP, astrocyte marker; NeuN, neuron marker; IBA-1, microglia marker) (Green) (A) or TLR7 (Red). GFAP and IBA-1 staining in cerebral cortex; NeuN staining in hippocampus. The presentative images were acquired using fluorescence microscopy (B). Bar = 20 μm. (**C**) Neonatal WT mice were intracranially injected with PBS or EV71. The cerebral cortex sections from mice after 3 days EV71 incubation were subjected to immunostaining with DAPI (Blue), neural cells markers (Green) and dsRNA (Red). The presentative images were acquired using fluorescence confocal microscopy. Bar = 20 μm. (**D** and **E**) Neonatal WT and TLR7^-/-^ mice were intracranially injected with PBS or EV71. After 3 days EV71 incubation, brain sections from mice were subjected to immunostaining with DAPI (Blue), GFAP (Green) and IL-6 (Red) (D). Bar = 20 μm. Cells were observed using fluorescence confocal microscopy and the IL-6 expression in GFAP-positive (GFAP^+^) cells was calculated (E). Graphs show mean ± SD. **, *P*<0.01. (**F** and **G**) There-day-old WT mice were intracranially injected with 10 μl PBS or 10 μl PBS containing 50 μg R837 per mouse, and then sacrificed on day 1 post R837 administration. Immunostaining of the brain’s cortex sections from day 1 was probed with IL-6 (Red), GFAP (Green) and stained with DAPI (Blue) (F), or cl-Caspase-3 (Red), GFAP (Green) and DAPI (Blue) (G). The presentative images were acquired using fluorescence microscopy. Bar = 20 μm.

As TLR7 is expressed in the cortex and hippocampus, it regulates neural development and brain function even in the absence of infectious or pathogenic molecules [11, 28]. We first evaluated the distribution of neural cells in the cerebral cortex or hippocampal areas of WT and TLR7^-/-^ mice based on neural markers. Immunohistochemical analysis revealed that there were no significant differences in numbers and distributions of astrocytes (GFAP-positive), neurons (NeuN-positive), or microglia (IBA-1 positive) between cerebral cortexes or hippocampal areas of WT and TLR7^-/-^ mice (Fig 6A). Notably, confocal microscopy analyses revealed that TLR7 localized with GFAP, NeuN, and IBA-1 in the cerebral cortex of WT mice, and TLR7 was not detected in cerebral cortex of TLR7^-/-^ mice as expected (Fig 6B), suggesting that TLR7 is expressed in astrocytes, neurons, and microglia in the murine cerebral cortex.

As EV71 antigen has been detected in neurons and astrocytes in CNS tissues of infected rhesus macaques and human patients [29], we further assessed where EV71 replicates in cerebral cortex of mice. Confocal microscopy analyses indicated that the viral dsRNA mainly localized with GFAP, rather than with NeuN or IBA-1 (Fig 6C), suggesting that EV71 preferentially replicates in astrocytes of the murine cerebral cortex. In addition, the localization and expression of endogenous IL-6 protein in the cerebral cortex upon EV71 infection were examined. In the absence of infection, endogenous IL-6 protein was barely detected in the cerebral cortex of WT or TLR7^-/-^ mice, whereas upon EV71 infection IL-6 levels were significantly elevated in the cerebral cortex of WT mice relative to TLR7^-/-^ mice (Fig 6D and 6E, and S7 Fig). Furthermore, EV71-induced IL-6 mainly localized in GFAP positive cells (Fig 6D and 6E), moderately in IBA-1 positive cells (S7A and S7B Fig), while marginally in NeuN positive cells (S7C and S7C Fig).

EV71 preferentially infects and induces IL-6 production in astrocytes. Thus, we would like to figure out how TLR7 mediated astrocytic cell apoptosis with IL-6 production. The neonatal mice were intracranially injected with TLR7 agonist R837. Immunofluorescence staining results revealed that IL-6 production (Fig 6F) and Caspase-3 cleavage (cl-Caspase-3) (Fig 6F) were induced in GFAP positive cells in cerebral cortex of mice after R837 administration for 1 day. While these inductions were observed continuously in cerebral cortex of mice from 3 to 5 days upon R837 administration (S8A and S8B Fig). IL-6 protein was consistently induced in CSF of mice after R837 administration from 1, 3, to 5 days (S8C Fig). Altogether, these results confirmed that EV71 preferentially infects and induces IL-6 production through TLR7 in brain astrocytes in mice.

### TLR7 promotes IL-6 production and astrocytic cell apoptosis upon EV71 infection

**Fig 7:**
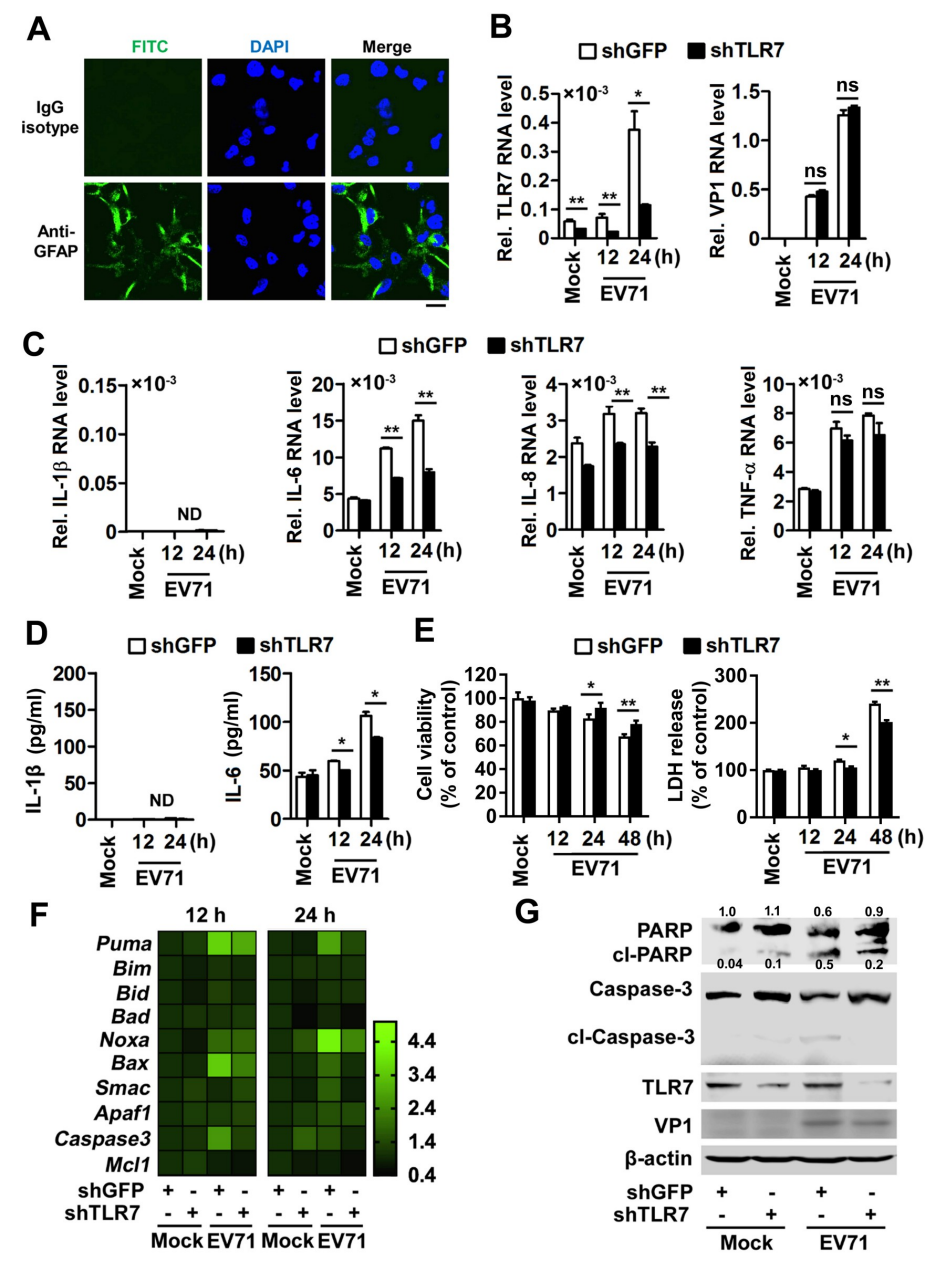
TLR7 promotes IL-6 production and astrocytic cell apoptosis upon EV71 infection. (**A**) U251 cells were seeded on 20-mm cover slips and probed with mouse anti-GFAP or isotype IgG antibodies (Green) and DAPI (Blue). The cells were visualized by confocal laser scanning microscopy. Bar = 20 μm. (**B**–**G**) U251 cells (2×10^6^) were transfected with 2 μg plasmid transcribing siRNA specific to *TLR7* (shTLR7) or its control (shGFP) and then mock-infected or infected with EV71 (MOI = 0.5) for 12 or 24 h, respectively. The total mRNA of treated cells was extracted. *TLR7* mRNA and EV71 *VP1* RNA (B) as well as cytokines mRNA levels (C) were determined by qPCR. The supernatants of treated cells were collected and IL-1β and IL-6 protein levels were measured by ELISA (D). Cell viability and LDH release of treated cells for 12, 24 or 48 h were examined using CCK8 and LDH assay, respectively (E). Data are shown as mean ± SD and correspond to a representative experiment out of three performed. ns, non-significant; *, *P* < 0.05; **, *P* < 0.01. The RNA was extracted from the transfected cells at 12 or 24 h post EV71 infection. The levels of apoptosis-associated genes mRNAs were measured by qPCR (F). Data are shown as fold changes of RNA expression compared to mock samples. The protein was extracted from the transfected cells at 24 h post EV71 infection and then detected by Western blotting with targeted antibodies (G). Relative PARP protein expression to internal control is quantified using Image J software.

Given that EV71 preferentially infects mice astrocytes and induces neuropathogenesis *via* TLR7, we thus further assessed the role of TLR7 in promoting cell apoptosis upon EV71 infection in human astroglioma U251 cells. Our results confirmed that GFAP (an astrocyte marker) was highly expressed in the cytoplasm of U251 cells (Fig 7A). Firstly, the dynamic EV71 replication in U251 cells was estimated. We found the levels of viral RNA (S9A Fig) and proteins (S9B Fig) were continuously increased ranged from 0 to 48 h, while slightly reduced at 60 h post-infection in U251 cells. The role of TLR7 in regulating cell apoptosis in U251 cells upon EV71 infection was then evaluated using shRNAs specifically targeting TLR7 (shTLR7) or shGFP (as a negative control). qPCR assays revealed that *TLR7* mRNA expressed in mock-infected cells or induced in EV71-infected cells was significantly attenuated by shTLR7 (Fig 7B, left), indicating that shTLR7 is highly efficient at achieving knockdown in cells. The results also indicated that EV71 *VP1* RNA was not affected by shTLR7 (Fig 7B, right), suggesting that TLR7 doesn’t influence EV71 replication in U251 cells. Notably, *IL-1β* mRNA was barely detected in U251 cells upon EV71 infection, whereas *IL-6* and *TNF-α* mRNA was highly expressed and significantly induced by EV71 in these cells (Fig 7C), while the inductions of *IL-6* and *IL-8* mRNA were significantly down-regulated by shTLR7, whereas *TNF-α* mRNA was not affected by shTLR7 (Fig 7C). In addition, ELISA assays further demonstrated that IL-1β protein level was not detectable in U251 cells (Fig 7D, left), whereas there was abundant IL-6 production and prominently induced by EV71 in these cells, and this induction was decreased by shTLR7 (Fig 7D, right). In parallel, IL-8 protein level was mildly elevated by EV71, but this production was not affected by shTLR7 (S9C Fig). These results suggest that IL-8 slightly secreted from astrocytes upon viral infection independent on TLR7, although IL-8 protein can consistently release from astrocytes [30]. Collectively, these results suggest that TLR7 facilitates IL-6 expression and production upon EV71 infection in U251 cells.

Next, the role of TLR7 in mediating EV71-induced neurotoxicity was assessed. Upon EV71 infection, cell viability was reduced in the presence of shGFP, whereas this reduction was reversed in the presence of shTLR7 (Fig 7E, left), suggesting TLR7 is involved in mediating EV71-induced cell death in U251 cells. In addition, upon EV71 infection, the lactate dehydrogenase (LDH) release was induced in the presence of shGFP, whereas such induction was significantly attenuated in the presence of shTLR7 (Fig 7E, right), further indicating that TLR7 is involved in promoting the EV71-induced death in U251 cells. Since TLR7 and TLR8 are known to be functionally related [31], the biological relationship in functionality of TLR7 with TLR8 was also investigated. TLR8 was expressed in astrocytes of both WT and TLR7^-/-^ mice cerebral cortex (S10A Fig), indicating that TLR7 deficiency doesn’t affect TLR8 expression in astrocytes of mice cerebral cortex. Then, siRNA silencing of TLR8 was applied in human U251 cells, followed by stimulated with R837 (TLR7 but not TLR8 agonist) as a positive control and EV71, revealing no effect on TLR7 expression after TLR8 silencing (S10B Fig). Upon R837 or EV71 treatment, cell viability was reduced while LDH release was induced (S10C Fig). However, these changes were not affected by knockdown of TLR8. These results further indicated EV71-induced neurotoxicity in human U251 cells might rely on TLR7 but not TLR8. In contrast, upon VTX-2337 (TLR8 but not TLR7 agonist) stimulation, cell apoptosis was strongly induced (S10D Fig). In parallel, cell viability was reduced while LDH release was induced (S10E Fig). Therefore, the data reveal that EV71 activates TLR7 signaling and IL-6 production independent of TLR8 in astrocytes.

Furthermore, the TLR7-mediated astrocytic cell apoptosis upon EV71 infection was explored. The qPCR analyses revealed that upon EV71 infection, the mRNA levels of apoptotic initiators including *Puma*, *Bad*, *Noxa*, and *Bax*, were induced in shGFP-transfected cells, whereas this upregulation was attenuated in shTLR7-transfected cells (Fig 7F). Moreover, immunoblotting for cleaved-poly(ADP-ribose) polymerase (PARP) and cleaved-Caspase-3 revealed that upon EV71 infection, PARP cleavage and Caspase-3 cleavage were induced in shGFP-transfected cells, whereas these cleavage events were reduced in shTLR7-transfected cells (Fig 7G). Therefore, these results suggest that TLR7 facilitates astrocytic cell apoptosis upon EV71 infection. Taken together, our results thus reveal that TLR7 promotes IL-6 production to facilitate astrocytic cell apoptosis upon EV71 infection.

### IL-6 blockade attenuates astrocytic cell apoptosis upon EV71 infection in U251 cells

We further assessed that TLR7 mediated astrocytic cell apoptosis with IL-6 production. In U251 cells, upon R837 treatment, cell viability was reduced (S11A Fig) while LDH release was induced (S11B Fig). Moreover, similar with EV71 infection, R837 stimulation induced IL-6 production (S11C Fig), and the cleavage of PARP and Caspase-3 (S11D Fig) in U251 cells. The data demonstrated that TLR7 agonist caused IL-6 expression and apoptosis in U251 cells (*in vitro*). In addition, we found that an appropriate concentration of IL-6 (ranged from 20 to 500 pg/ml) activated Caspase-3 cleavage in U251 cells (S11E and S11F Fig), suggesting that TLR7-mediated IL-6 production triggers astrocytic cell apoptosis.

**Fig 8:**
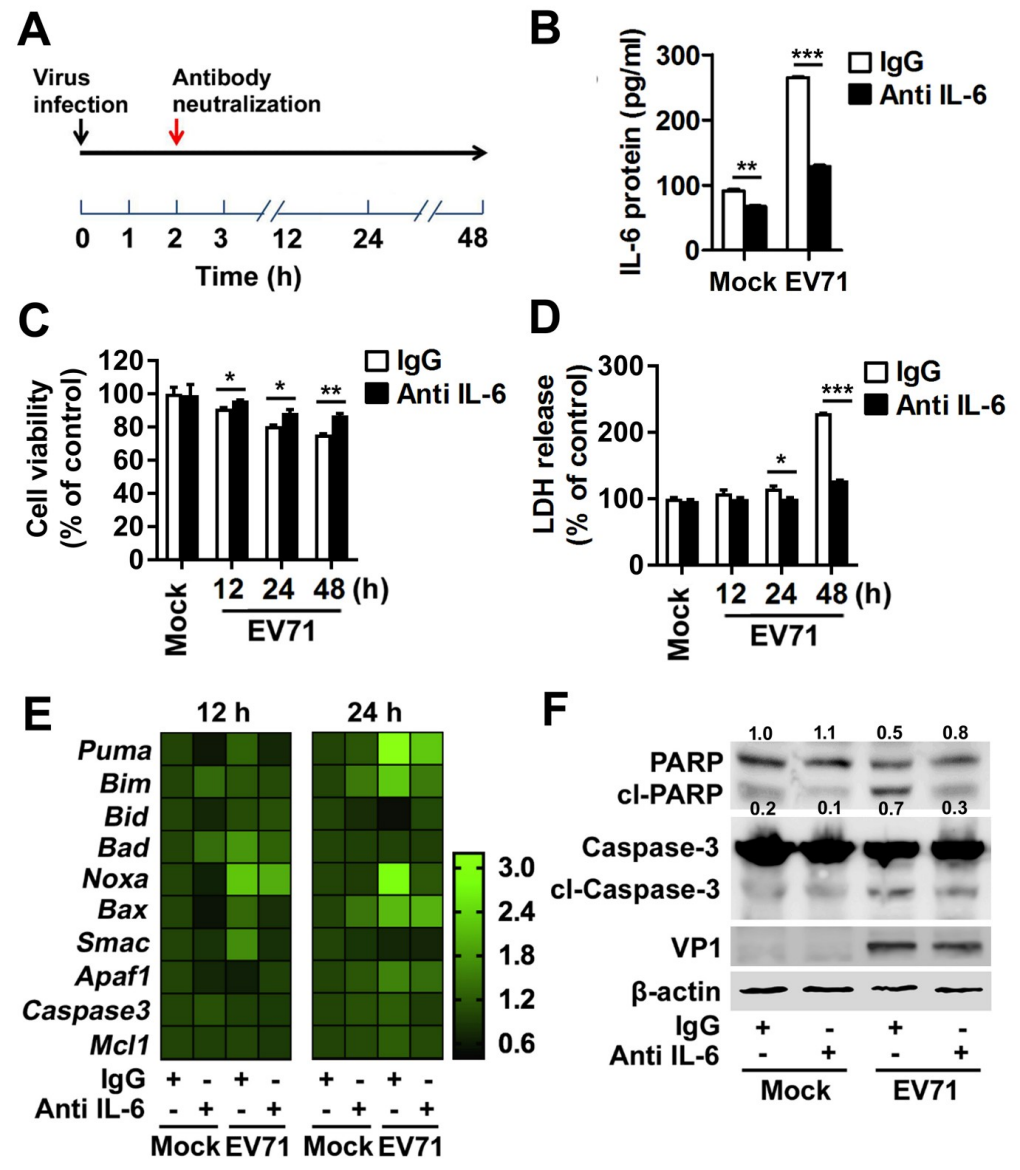
IL-6 blockade attenuates astrocytic cell apoptosis upon EV71 infection in U251 cells. (**A**) The schematic of IL-6 antibody neutralization upon EV71 infection. 2×10^6^ U251 cells were blocked with neutralized mouse anti-human IL-6 antibody (0.5 μg/ml) or mouse isotype IgG (0.5 μg/ml) at 2 h post EV71 infection (MOI = 0.5). (**B**) The supernatants of treated cells were collected and IL-6 protein level was measured by ELISA. (**C** and **D**) Cell viability (C) and LDH release (D) of treated cells for 12, 24 or 48 h were examined using CCK8 and LDH assay, respectively. Data are shown as mean ± SD and correspond to a representative experiment out of three performed. ns, non-significant; *, *P* < 0.05; **, *P* < 0.01; ***, *P* < 0.001. (**E**) The RNA was extracted from the transfected cells at 12 or 24 h post EV71 infection. The levels of apoptosis-associated genes mRNAs were measured by qPCR. Data are shown as fold changes of RNA expression compared to mock samples. (**F**) The protein was extracted from the transfected cells at 24 h post EV71 infection and then detected by Western blotting with targeted antibodies. Relative PARP protein expression to internal control is quantified using Image J software.

As TLR7 promotes IL-6 production and cell apoptosis induction by EV71 infection in U251 cells, we further explored the role of IL-6 in TLR7-mediated cell apoptosis induced by EV71. U251 cells were infected with EV71 and then treated with isotype IgG (IgG) or anti-IL-6 antibody (IL-6-Ab) (Fig 8A). ELISA results revealed that IL-6 protein levels were sharply reduced by IL-6-Ab treatment in cell supernatants of both mock-infected or EV71-infected cells (Fig 8B), demonstrating that the IL-6-Ab blockade is efficient. Upon EV71 infection, cell viability was attenuated in the presence of IgG, whereas this reduction was reversed by IL-6-Ab (Fig 8C). In addition, LDH release was increased in the presence of IgG, whereas this increase was reduced by IL-6-Ab (Fig 8D). Moreover, upon EV71 infection, the mRNA levels of the apoptotic initiators *Puma*, *Bad*, *NoxA*, and *Bax* were enhanced in IgG-treated cells, whereas these enhancements were reduced in IL-6-Ab-treated cells (Fig 8E). Notably, upon EV71 infection, PARP cleavage and Caspase-3 cleavage were increased in IgG-treated cells, whereas these cleavage events occurred less frequently in IL-6-Ab-treated cells (Fig 8F). Collectively, these results demonstrate that IL-6 is involved in promoting TLR7-mediated cell death in U251 cells upon EV71 infection.

### TLR7 upregulation, IL-6 induction and astrocytic cell apoptosis in EV71-infected human brain

**Fig 9:**
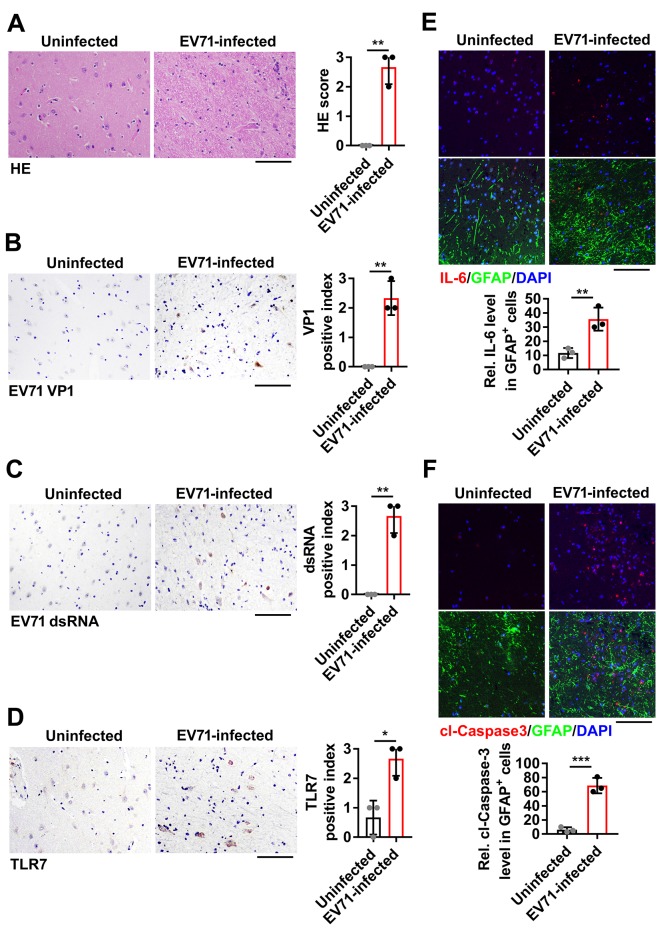
Histopathological characteristics in brain tissues from EV71-infected human. (**A**) The hematoxylin-eosin (H&E) staining of cerebral cortex sections from human brain tissues (uninfected and EV71-infected; each group, n = 3). The presentative images were acquired using light microscopy. Bar = 100 μm. (**B**–**D**) Cerebral cortex sections from EV71-infected or uninfected human were subjected to IHC staining with anti-EV71 VP1 antibody (B), anti-dsRNA antibody (C) or anti-TLR7 antibody (D). The presentative images were acquired using light microscopy. Bar = 100 μm. The relative expression of indicated was shown as a positive index and quantified with Image J software. (**E** and **F**) Human brain tissue sections were fixed and stained with GFAP (Green) and IL-6 (Red) (E) or cl-Caspase-3 (Red) (F). The presentative images were acquired using fluorescence microscopy. Bar = 100 μm. The relative expression of IL-6 or cl-Caspase-3 in GFAP positive (GFAP^+^) cells was calculated with Image J software. Data are shown as mean ± SD. *, *P* < 0.05; **, *P* < 0.01; ***, *P* < 0.001.

EV71 is neurotropic that has been demonstrated in human and primate pathological specimens, and the viral distribution is distinct and stereotyped [9]. To extrapolate the role of TLR7 in EV71-induced neuropathogenesis to human infections, we performed the histological analysis in six human brain specimens. The pathological changes were characterized as inflammatory cell infiltration and neurodegeneration in both cerebral cortex (Fig 9A) and cerebellum (S12A Fig) of EV71-infected death cases. We also observed that the EV71 antigen VP1 was expressed in the cerebral cortex (Fig 9B) and cerebellum (S12B Fig) of EV71-infected human brain tissues. The similar observations were found that EV71 dsRNA was distributed in EV71-infected human brain regions, including the cerebral cortex (Fig 9C) and cerebellum (S12C Fig). In the parallel histological analysis, TLR7 expression appeared at a higher level in the cerebral cortex of EV71-infected human brain tissues than uninfected ones (Fig 9D). The similar phenomenon was observed in the cerebellum of EV71-infected human brain specimens (S12D Fig). Further histological analysis revealed the IL-6 expression (Fig 9E), as well as cl-Caspase-3 level (Fig 9F) in GFAP positive cells were obviously upregulated in EV71-infected human cerebral cortex. In addition, these up-regulations of the IL-6 expression (S12E Fig), and cl-Caspase-3 level (S12F Fig) in GFAP positive cells were displayed in EV71-infected human cerebellum. However, we noticed that the up-regulations of the IL-6 and cl-Caspase-3 expression in the cerebellum were less than that in the cerebral cortex of EV71-infected human brain tissues, which is consistent with those found in EV71-infected mice brain. Altogether, these data demonstrated that TLR7 upregulation, IL-6 induction and astrocytic cell apoptosis occurred in human brain upon EV71 infection.

**Fig 10:**
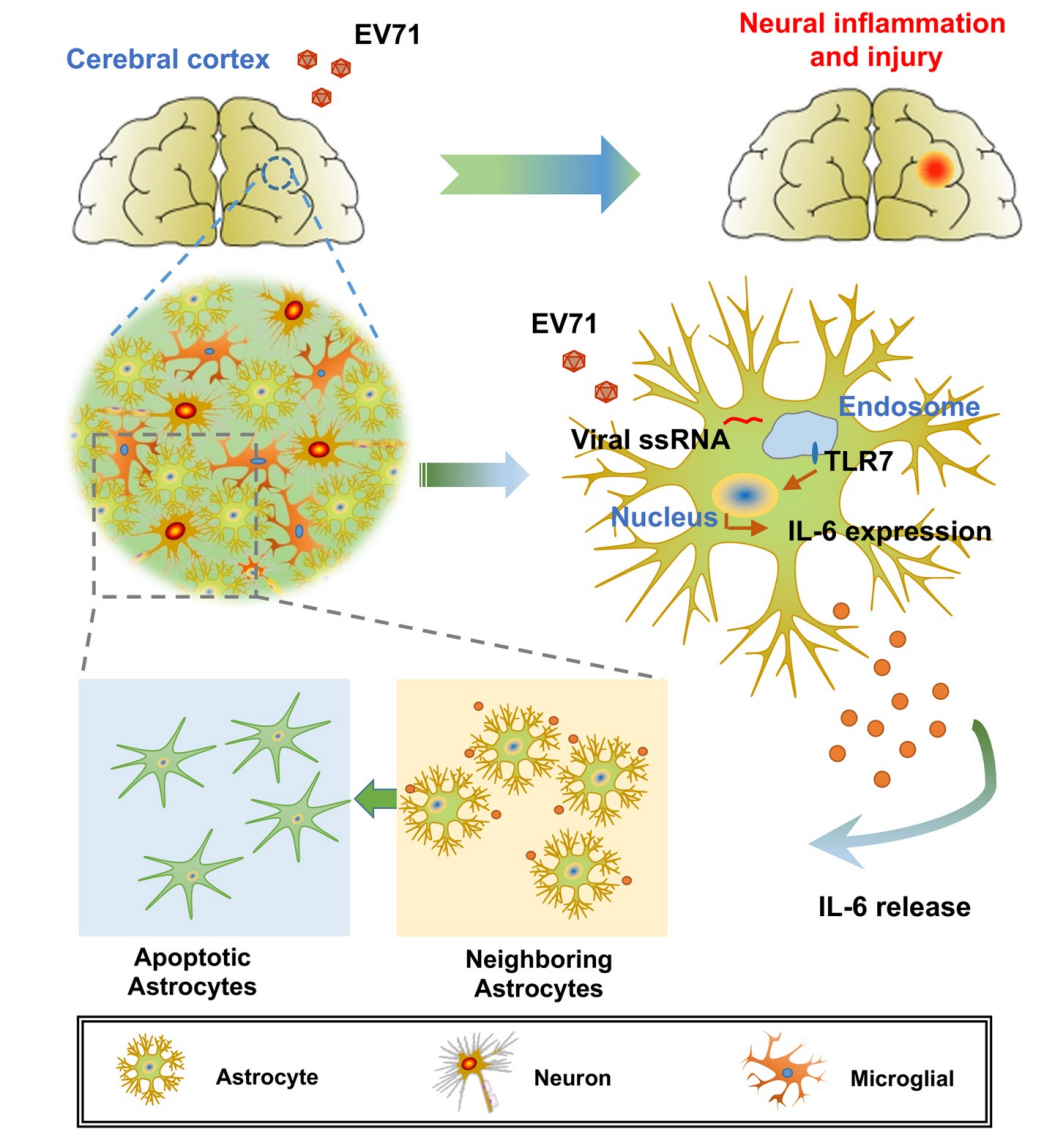
A proposed mechanism underlying the regulation brain pathogenesis upon EV71 infection. The neurotropic EV71 preferentially infects and replicates in astrocytes of mice and human brain cerebral cortex. EV71 ssRNA is then recognized by TLR7 to trigger the signaling events, which in turn dominantly promote IL-6 protein production in astrocytes. The release of IL-6 protein in EV71-infected cells affects astrocyte self and neighboring astrocytes, resulting in the induction of cell apoptosis. The dysfunctional astrocytes disrupt brain homeostasis, which systematically develops neural inflammation and injury in brain.

Taken together, we propose that the neurotropic EV71 preferentially infects and replicates in astrocytes of the murine and human cerebral cortex. EV71 RNA is recognized by TLR7 to trigger the signaling events and to promote IL-6 production and release, which results in the induction of cell apoptosis and in the development of neuroinflammation and brain injury (Fig 10).

## Discussion

Toll-like receptors (TLRs) act as innate immune receptors to recognize conserved pathogen-associated molecules in certain immune cell types. Various TLRs are also expressed in neurons, astrocytes, and in microglial cells, and they are conducive to the immunological responses of the CNS [32–34]. Emerging evidence suggests that TLRs play fundamental roles in brain development and homeostasis [28, 35, 36]. TLR7 is widely expressed in immune cells, intestinal cells, lung cells, and neural cells, wherein it is also able to detect and respond to specific viral RNAs, single-stranded oligoribonucleotides, siRNAs, and microRNAs, leading to intracellular signaling pathway activation and cytokine production [10, 11, 19, 37]. In various forms of non-infectious CNS injury, TLR7 also serves as a prominent mediator of neurodegeneration [11, 14, 38]. TLR7 can thus play broad regulatory roles across a range of pathways pertaining to neurodegeneration, innate immunity, and cell biology.

We have previously shown that TLR7 orchestrates immune and inflammatory responses in macrophages during EV71 infection [19]. Given the neurotropic pathology associated with EV71, we speculated that TLR7 may regulate the immunological protection of CNS upon EV71 infection. Using intracranial infection model which is suitable for evaluating EV71 neurovirulence [4], the neurotropic EV71 strain was intracranially injected in 3-day-old neonatal WT and TLR7^-/-^ mice. Compared with WT mice, TLR7^-/-^ mice were protected from EV71-induced weight loss, hind-limb paralysis, and lethality. Further histological analyses revealed a reduction in EV71-induced neurodegeneration in TLR7^-/-^ mice, suggesting TLR7 was essential for EV71 neurovirulence in mice. Unexpectedly, further findings revealed EV71 replication and tissue distribution were unaffected in the cerebral cortex of WT and TLR7^-/-^ mice. This suggested that TLR7 failed to act as an antiviral immune receptor in these tissues, instead of serving as a death receptor in the context of murine brain neuropathogenesis upon EV71 infection. Thus, we uncovered an additional biological function of TLR7 in the context of EV71-induced neurological lesion-related symptoms in the brains of mice. TLR3, TLR7, and TLR9 are known to be important host factors involved in restricting EV71 infection in diverse infection models *in vivo* and *in vitro* [19, 39, 40]. Given TLR7 systemically modulates immune response, it should be emphasized that the different roles of TLR7 on EV71 infection might result from specifically viral distribution in mice organs in distinct EV71 infection models, such as oral and intraperitoneal routes [39, 41]. In this study, we found viral distribution in a high level in cerebral cortex of the intracranially EV71-infected mice, which is similar with the EV71-infected human brain. Thus, it is ideal to investigate the function of TLR7 in EV71-induced neuropathogenesis in the intracranial infection model.

In the CNS, the TLR7-mediated apoptosis pathway leads to cell death under either physiological (eg. neurodegeneration) or pathological (eg. neurotropic virus infection) conditions [11,12,42]. To investigate the role of TLR7 in EV71-related neurological damage, we therefore detected multiple markers of apoptosis including activated Caspase-3, DNA damage, and apoptosis-associated gene expression in the cerebral cortex of WT and TLR7^-/-^ mice. Our observations revealed that the induction of apoptosis upon EV71-infection was significantly reduced in TLR7^-/-^ mice. The coordinated neuroinflammatory response may be an important factor in neural injury [24]. In TLR7-mediated neural apoptosis upon EV71-infection, the level of IL-6 was predominantly induced in the cerebral cortex on days 3 and 5 after EV71 infection, and was also specifically modulated by TLR7. Subsequently, we assessed the role of IL-6 in TLR7-mediated neuropathogenesis upon EV71 infection. In an intracranial injection model, IL-6 blockade effectively protected EV71-infected mice from neurological lesion-related symptoms. Correspondingly, IL-6 blockade significantly reduced the induction of apoptosis in the cerebral cortex of EV71-infected mice, while IL-6 blockade mediated such protection is independent of the virus load. Similar with findings regarding that IL-6 blockade protects mice from EV71-induced immunopathogenesis in the intraperitoneal infection model [43]. Besides the TLR/TNFα/apoptosis/necroptosis pathway as a driver of neurodegeneration in Alzheimer’s disease [11, 44], we identified a distinct TLR7/IL-6/apoptosis axis in viral neural pathogenesis mediated by TLR7 upon EV71 infection *in vivo*.

Neuroinflammation has been described in various neural cells of the brain, including astrocytes, neurons, and microglia, which contribute to disease development and progression [45]. As TLR7 protein is expressed in the cortex and hippocampus and regulates neural development and brain function even under non-infectious conditions [11, 28], we evaluated the viral distribution of neural cells in cerebral cortex of mice. Confocal microscopy revealed that viral dsRNA mainly colocalized with GFAP, rather than with NeuN or IBA-1, suggesting that EV71 preferentially infects and replicates in astrocytes of the murine cerebral cortex. Moreover, endogenously induced IL-6 protein mainly localized to astrocytes in the cerebral cortex of mice upon EV71 infection. In fact, EV71 antigen has primarily been detected in the neurons and astrocytes in CNS specimen from infected rhesus macaques and human patients [29]. Our data provide novel insights into the fact that EV71 preferentially infects and induces IL-6 in the astrocytes of mice, providing a novel explanation for the observed viral neuropathogenesis.

Astrocytes (also known as astroglia) are a class of neural cells of ectodermal and neuroepithelial origin that maintain homeostasis and provide for the defense of the CNS [46]. They are emerging as pivotal regulators of inflammatory responses in the context of CNS injury [47,48]. Given that EV71 preferentially infected astrocytes, we further evaluated the role of TLR7 in the human astroglioma U251 cells upon EV71 infection. Using TLR7 silencing and IL-6 neutralization approaches, we confirmed that TLR7 knockdown and IL-6 blockade attenuated astrocytic cell apoptosis upon EV71 infection. Astrocytes are tightly integrated into neural networks and act within the context of neural tissue *in vivo*, and therefore the neurotoxic reactive astrocytes are also induced by activated microglia or damaged neurons in neurodegenerative diseases [49, 50]. In this study, we propose that EV71 RNA triggers TLR7 signaling, subsequently promoting IL-6 expression primarily in astrocytes. The release of IL-6 protein by these EV71-infected cells mediate autocrine and paracrine signaling in proximal astrocytes, leading to astrocytic cell apoptosis. These dysfunctional astrocytes disrupt brain homeostasis, leading to the systematic development of neural inflammation and injury in brain, and even causing death in the case of some EV71 infections. However, the interplay of EV71-infected astrocytes with other neural cell types such as microglia or neurons involved in viral neural pathogenesis remains to be further studied. Altogether, these data reveal that astrocytes are responsible for EV71-induced neurodegeneration, a potential role of microglia in such pathology needs to clarify beyond this limited study. As such, new approaches, including human brain organoids, and 3D microfluidic platforms, have major advantages in providing a micro-physiological system more closely reflecting the *in vivo* brain environment [51], which are ideal to understand astrocytes-microglia or neurons crosstalk in EV71-associated CNS disorders.

Viral infections of the CNS can manifest in various forms of inflammation to influence the CNS homeostasis, including tick-borne encephalitis virus (TBEV), Zika virus (ZIKV), West Nile virus (WNV), and Japanese encephalitis virus (JEV) [42, 52, 53], which has led to substantial focus on the responses of astrocytes in the context of CNS injury. Here we further expand current understanding of not only the mechanism underlying TLR7-mediated neural pathogenesis involved in IL-6 induction, but also the effects of IL-6 on the fate of astrocytes in the brain upon EV71 infection. Our findings may thus provide clues to aid the design of new strategies aimed at better containing and mitigating neurotropic virus- and astrocyte- dependent neural pathogenesis.

## Materials and methods

### Animal studies

C57BL/6 WT mice were purchased from Shanghai Laboratory Animal Center. The TLR7^-/-^ mice were originally obtained from The Jackson Laboratory (Bar Harbor, Maine, USA). The mice were housed under specific pathogen-free (SPF) conditions in individually ventilated cages. Three-day-old suckling mice were intracranially injected with 10 μl PBS containing 1×10^9^ plaque-forming units (PFU)/ml of EV71 with a 50-μl gastight microsyringe (Hamilton, Reno, NV, USA). Following EV71 infection, mouse weights and clinical scores were recorded every day until one-week post-treatment. The clinical scores were defined as follows: 0, healthy; 1, ruffled hair and hunched back; 2, limb weakness; 3, paralysis in one limb; 4, paralysis in both limbs; and 5, death.

For intracranial IL-6 neutralization, neonatal WT mice were intracranially injected with 10 μl PBS or EV71 per mouse. Eight hours later, the mice were divided into groups and separately intracranially injected with IgG isotype or anti-IL-6 at a dose of 8 μg/per mouse, and this injection was repeated the following day. For R837 administration *in vivo*, the suckling mice were intracranially injected with 10 μl PBS containing R837 (TLR7 agonist, Catalog number: HY-B0180; MedchemExpress; NJ, USA) at a dose of 50 μg/per mouse.

To collect cerebrospinal fluid (CSF), there-day-old WT mice were sacrificed and removed the entire spine of the mouse, 10 μl of CSF was aspirated with microsyringe, and then diluted in 1ml PBS. Finally, 200 μl diluted CSF was subjected to ELISA assay to detect IL-6 protein expression using mouse IL-6 ELISA kit (4A Biotech Co. Ltd; Beijing, China), or RNA extraction to measure EV71 RNA copies.

### Ethics statement

All animal studies were performed in accordance with the Guide for the Care and Use of Laboratory Animals published by the US National Institutes of Health (NIH Publication No. 85–23, revised in 1996). All procedures involving mice and experimental protocols were approved by the Institutional Animal Care and Use Committee (IACUC) of the College of Life Sciences, Wuhan University (Permit numbers: 2017–016).

### Human brain tissues

Brain tissue samples from six deceased patients (three were confirmed to be EV71-infected and the other three were uninfected as the negative controls) were obtained from the School of Forensic Medicine, Kunming Medical University. The tissues were fixed in formalin, and embedded in paraffin. The infected group included two males and one female, and the average age was 27.3 months. The average age of control group was 16 months (Table 2).

**Table 2:** Demographic and baseline characteristics of EV71-negative individuals and EV71-positive patients^a^.

Number	Age (month)	Gender	EV71 infection^*b*^
1	8	Male	-
2	35	Female	-
3	5	Male	-
4	28	Male	+
5	36	Male	+
6	18	Female	+

^*a*^ All EV71-positive individuals were confirmed to be negative for other enteroviruses and were not suffering from any concomitant illness, did not show any serological markers suggestive of autoimmune disease. Matched by sex and age, EV71-negative individuals with no history of HFMD were randomly selected as controls.

^*b*^ The status of EV71 infection is marked as “-” and “+” for EV71-negative individuals and EV71-positive patients, respectively.

### Cell lines and transfection

Human astroglioma U251 cells were purchased from American Type Culture Collection (ATCC) (Manassas, VA, USA). Human embryonal rhabdomyosarcoma (RD) cells were obtained from the China Center for Type Culture Collection (CCTCC) (Wuhan, China). All cell lines were cultured in Dulbecco’s Modified Eagle Medium (DMEM) (Invitrogen, Carlsbad, CA), supplemented with 10% fetal bovine serum (FBS; Gibco, Grand Island, NY), 100 U/ml penicillin, and 100 mg/ml streptomycin sulfate at 37°C in a 5% CO_2_ incubator.

For cell transfection, U251 cells (2×10^6^) were cultured in 6-plate well in 2 ml medium, and then 2 μg plasmids transcribing shRNA were transiently transfected into cells using Lipofectamine 2000 (Invitrogen) according to the manufacturer's instructions.

### Virus and infection

The neurotropic Enterovirus 71 (EV71) strain (Xiangyang-Hubei-09) was originated from the brain tissue of a one-year-old dead EV71-infected infant in Xiangyang city, Hubei province in China, and isolated previously in our laboratory (GenBank accession no. JN230523.1). The virus stock was propagated in RD cells, and virus titration and inoculation with EV71 were performed as described previously [54,55]. The inactivated EV71 virus was obtained in two forms: (1) the irradiation by ultraviolet (UV) lamp for 2 h; (2) heating in water at 65°C for 30 min.

Aliquots were stored at -80°C prior to usage. *In vitro*, U251 cells were infected with the virus at the indicated multiplicities of infection (MOIs) and unbound virus was washed away 2 h later, after which samples were incubated at 37°C for an additional 24 or 48 h.

### Reagents

Mouse anti-TLR7 (clone ID, 4G6) was purchased from Novus Biologicals (Littleton, CO, USA). Mouse anti-Neurofilament-L (DA2), as well as rabbit antibodies against Hexaribonucleotide binding protein-3 (NeuN) (clone ID, D4G40), Glial fibrillary acidic protein (GFAP) (clone ID, E4L7M), Caspase-3 (clone ID, D3R6Y), Cleaved Caspase-3 (Asp175) (clone ID, 5A1E), and Poly(ADP-ribose) polymerase (PARP) (clone ID, 46D11) were purchased from Cell Signaling Technology (Beverly, MA, USA). Rabbit anti-Ionized calcium binding adaptor molecule 1 (IBA-1) was purchased from Abcam (Cambridge, United Kingdom). Mouse anti-EV71 VP1 was purchased from Abnova Company (Taiwan, China). Mouse anti-dsRNA monoclonal antibody J2 was obtained from Scicons (Hungary). Rabbit anti-TLR8 (abs102571) was obtained from Absin Bioscience Inc. (Shanghai, China). Rabbit anti-Glyceraldehyde-3-phosphate dehydrogenase (GAPDH) was purchased from ProteinTech Group (Chicago, IL, USA). Mouse anti-β-actin was purchased from Santa Cruz Biotechnology (Santa Cruz tech., CA, USA). Rat antibodies against mouse IL-6 (clone ID, MP5-20F3) or isotype IgG1 (clone ID, TNP6A7) were purchased from BioXcell (West Lebanon, NH, USA). A mouse monoclonal antibody against human/primate IL-6 (clone ID, #6708) and isotype IgG1 (clone ID, #11711) was purchased from R&D systems (Minneapolis, MN, USA). Recombinant human IL-6 protein (Catalog number: 200–06) was purchased from Pepro Tech Inc. (Rocky Hill, NJ, USA). Cell apoptosis inducer, etoposide (Catalog number: HY-13629), and TLR8 agonist, VTX-2337 (Catalog number: HY-13773) were purchased from MedchemExpress (NJ, USA).

The shTLR7 and shGFP plasmids expressing a short-hairpin RNA (shRNA) targeting TLR7 or Green fluorescent protein (GFP) as a negative control were generated by inserting the appropriate DNA fragments into the pSilencer 2.1-U6 neo vector (Ambion, Inc., Austin, TX, USA), respectively, as described previously [19]. The specific DNA fragments were synthesized by Tsingke Biological Technology (Beijing, China). The siRNA specific to the negative control (NC) and human TLR8 were synthesized by RiboBio (Guangzhou, China) and used at the concentration of 50 nM. siRNA-NC targeted the sequence 5’-TTCTCCGAACGTGTCACGT-3’, siRNA-TLR8 targeted the sequence 5’-GATGGTGGTGCTTCAATTA-3’.

### Immunohistochemistry and immunofluorescence staining

Tissue samples were mounted on slides from paraffin blocks (5-μm sections), deparaffinized three times in xylene for 5 min, and hydrated in a methanol gradient (100%, 95%, 70%, and 50%). 3% H_2_O_2_ and 10 mM citrate buffer (pH6) was used for antigen retrieval. Nonspecific peroxidase activity was blocked for 30 min with 5% bovine serum albumin (BSA). The slides were then incubated with the primary antibody overnight at 4°C and then washed with PBS for 10 min. The biotinylated secondary antibody was initially applied for 30 min, after which an avidin biotin complex kit (Dako/Agilent Technologies, Santa Clara, CA, USA) was used for an additional 30 min. 3,3’-diaminobenzidine tetrahydrochloride hydrate (DAB) with 5% H_2_O_2_ was used for detection. Slides were then counterstained with hematoxylin and eosin (H&E) to stain nuclei. Immunohistochemistry was conducted at each site using specific antibodies, and samples were visualized by microscopy (Olympus, Tokyo, Japan).

For immunohistochemistry analysis, the IHC reaction was assessed using light microscopy following scoring criteria: 0, no or faint staining intensity; 1+, faint cytoplasmic staining; 2+, moderate and incomplete membranous staining; and 3+, strong membranous staining. The intensity of immunohistochemistry or immunofluorescence staining was generated using Image J software from three representative images.

### RNA extraction and quantitative PCR

RNA was extracted from homogenized mouse brain tissues or cultured cells using the TRIzol reagent (Invitrogen, Carlsbad, CA, USA). Quantitative real-time PCR (qPCR) analyses were performed using the Roche LightCycler 480 (Roche Diagnostics, Indianapolis, IN, USA) and SYBR RT-PCR kits (Roche) according to the manufacturer's instructions. The data represent absolute mRNA copy numbers normalized to GAPDH used as a reference gene. Relative fold expression values were determined by using the ^ΔΔ^Ct method. Primer sequences are listed in S1 Table.

### Western blotting

Radio-immunoprecipitation assay (RIPA) buffer containing protease inhibitors was used in order to lyse and extract protein from murine brain homogenates and cultured cells. Supernatant protein concentrations were then determined using a BCA assay kit, and protein lysates (100 μg) were resolved by SDS-PAGE and transferred onto nitrocellulose (NC) membranes (Amersham, Piscataway, NY, USA). Nonspecific binding was blocked with 5% nonfat dried milk (BD Biosciences, San Jose, CA, USA) for 1 h at room temperature. After three washes in PBS, the NC membranes were incubated with appropriate primary and secondary antibodies. Blots were then analyzed using a Luminescent Image Analyzer (Fujifilm LAS-4000; GE Life Sciences, Piscataway, NJ, USA). The relative target protein expression to internal control is quantified using Image J software.

### Enzyme-linked immunosorbent assays (ELISAs)

U251 cells (1×10^6^ per well) were seeded in 6-well culture plates with 10% FBS, and were transfected with shGFP or shTLR7 plasmids. At 24 h post-transfection, cells were infected with EV71 at a MOI = 0.5 for 2 h, and were then washed with PBS to remove the unbound virus. Then, cells were cultured in 2 ml of FBS-free DMEM for an additional 12 h. Finally, the cell supernatants were harvested after centrifugation at 10,000 *g* for 5 min for ELISAs. Supernatant cytokine levels were assessed using human IL-1β immunoassay (BD Biosciences, CA, USA), human IL-6 immunoassay kits (R&D systems, Minneapolis, MN, USA), and human IL-8 ELISA kit (4A Biotech Co. Ltd, Beijing, China) following the manufacturer’s provided instructions.

### Cell viability and LDH release assays

Cells (1×10^5^ per well) were seeded in 24-well plates after treatment. A cell viability assay was then performed using the Cell Counting Kit 8 (CCK8, Dojindo, Japan) according to the manufacturer's instructions. Cell injury was determined by measuring the lactate dehydrogenase (LDH) activity in FBS-free culture medium at 30 min after restoration using an LDH detection kit (Dojindo, Japan) according to the manufacturer’s instructions. Briefly, medium was harvested from a 24-well plate and centrifuged at 5000 rpm for 10 min. This cell-free supernatant was then incubated with the provided reaction mixture, and LDH activity was determined based on absorbance at 450 nm.

### Statistical analysis

All experiments were reproducible, and each set of experiments was repeated at least three times with similar results. Statistical significance for comparison of two means was assessed via unpaired Student’s *t-*test. Analyses were performed using GraphPad Prism 7 (San Diego, CA, USA). Survival curves were plotted using the Kaplan-Meier method, and significant differences in survival were calculated via the log-rank test. Data are means ± the standard deviation (SD), and statistical significance was evaluated using the following *P* values: *P* <0.05 (*), *P* <0.01 (**) or *P* <0.001 (***).

## Supporting information

S1 Fig Immunofluorescence analysis in cerebral cortex of intracranially EV71-infected mice. There-day-old WT mice were intracranially injected with 10 μl PBS, EV71-UV, EV71- Heated or EV71 per mouse (each group, n = 10–12) and sacrificed on day 1, 3 or 5 post-infection, respectively. (**A** and **B**) The EV71 virus RNA copies in cerebral cortex (A) or cerebellum (B) were determined by absolute quantitative PCR. Data are shown as mean ± SD. ***, *P* < 0.001. (**C** and **D**) The cerebral cortex sections of mice on day 3 post-infection from different groups were fixed and subjected to immunostaining with cl-Caspase-3 (Red), dsRNA (Green), and DAPI (Blue) (C). The presentative images were acquired using fluorescence microscopy. Bar = 20 μm. The relative expression of cl-Caspase-3 and dsRNA was quantified using Image J software (D). Data are shown as mean ± SD. (**E** and **F**) The cerebral cortex sections of mice on day 5 post-infection from different groups were immunostained with cl-Caspase-3 (Red), dsRNA (Green), and DAPI (Blue) (E). The presentative images were acquired using fluorescence microscopy. Bar = 20 μm. The relative expression of cl-Caspase-3 and dsRNA was quantified using Image J software (F). Data are shown as mean ± SD.
(TIF)

S2 Fig Immunofluorescence analysis in cerebellum of intracranially EV71-infected mice. There-day-old WT mice were intracranially injected with 10 μl PBS, EV71-UV, EV71- Heated or EV71 per mouse (each group, n = 10–12) and sacrificed on day 1, 3 or 5 post-infection, respectively. (**A** and **B**) The cerebellum sections of mice on day 3 post-infection from different groups were fixed and subjected to immunostaining with cl-Caspase-3 (Red), dsRNA (Green), and DAPI (Blue) (A). The presentative images were acquired using fluorescence microscopy. Bar = 20 μm. The relative expression of cl-Caspase-3 and dsRNA was quantified using Image J software (B). Data are shown as mean ± SD. (**C** and **D**) The cerebellum sections of mice on day 5 post-infection from different groups were immunostained with cl-Caspase-3 (Red), dsRNA (Green), and DAPI (Blue) (C). The presentative images were acquired using fluorescence microscopy. Bar = 20 μm. The relative expression of cl-Caspase-3 and dsRNA was quantified using Image J software (D). Data are shown as mean ± SD.
(TIF)

S3 Fig Distribution of EV71 in cerebral cortex and cerebellum of WT and TLR7^-/-^ mice. (**A** and **B**) WT mice and TLR7^-/-^ mice mock-infected or EV71-infected were sacrificed on 2, 3, 5, and 7 days post-infection (each group, n = 3–5). The mice cerebral cortex sections (A) and cerebellum sections (B) were fixed and subjected to IHC staining with EV71 VP1 antibody (Brown), respectively. The presentative images were acquired using light microscopy. Bar = 100 μm. EV71 VP1 relative expression was shown as VP1 positive index and quantified with Image J software. Data are shown as mean ± SD. ns, non-significant.
(TIF)

S4 Fig IL-6 protein production and EV71 load in different tissues of IL-6 Ab-treated mice. Neonatal WT mice were intracranially injected with 10 μl PBS or EV71 per mouse, and separately intracranially treated with IgG isotype or anti-IL-6 antibody. The different sections of mice on day 1 in different groups were subjected to IL-6 protein and EV71 load detection. (**A** and **B**) The proteins were extracted from individual mice cerebral cortex (A) or cerebellum (B) tissues and then the IL-6 protein level in tissues (per gram) was determined by ELISA assay. (**C** and **D**) IL-6 secretion in cerebrospinal fluid (CSF) (C) and peripheral blood (D) were determined by ELISA assay. (**E**-**H**) EV71 RNA was extracted from mice cerebral cortex (E), cerebellum (F), CSF (G) and peripheral blood (H). EV71 viral RNA copies were determined by absolute quantitative PCR. Data are shown as mean ± SD. ns, non-significant; *, *P* < 0.05; **, *P* < 0.01; ***, *P* < 0.001.
(TIF)

S5 Fig Immunofluorescence analysis of IL-6 and EV71 VP1 expression in cerebral cortex and cerebellum of IL-6 Ab-treated mice. Neonatal WT mice were intracranially injected with PBS or EV71 per mouse, and separately intracranially treated with IgG isotype or anti-IL-6 antibody. The cerebral cortex and cerebellum sections of mice on day 1 in different groups were immunostained with IL-6 (Red), EV71 VP1 (Green), and DAPI (Blue). (**A**) The presentative images of cerebral cortex sections were acquired using fluorescence microscopy. Bar = 20 μm. (**B**) The relative expression of IL-6 and EV71 VP1 in cerebral cortex was quantified using Image J software. (**C**) The presentative images of cerebellum sections were acquired using fluorescence microscopy. Bar = 20 μm. (**D**) The relative expression of IL-6 and EV71 VP1 in cerebellum was quantified using Image J software. Data are shown as mean ± SD. ns, non-significant; *, *P* < 0.05.
(TIF)

S6 Fig Immunofluorescence analysis of IL-6 and EV71 VP1 expression in spinal cord and skeletal muscle of IL-6 Ab-treated mice. Neonatal WT mice were intracranially injected with PBS or EV71 per mouse, and separately intracranially treated with IgG isotype or anti-IL-6 antibody. The spinal cord and skeletal muscle sections of mice on day 1 in different groups were immunostained with IL-6 (Red), EV71 VP1 (Green), and DAPI (Blue). (**A**) The presentative images of spinal cord sections were acquired using fluorescence microscopy. Bar = 20 μm. (**B**) The relative expression of IL-6 and EV71 VP1 in spinal cord was quantified using Image J software. (**C**) The presentative images of skeletal muscle sections were acquired using fluorescence microscopy. Bar = 20 μm. (**D**) The relative expression of IL-6 and EV71 VP1 in skeletal muscle was quantified using Image J software. Data are shown as mean ± SD. ns, non-significant; *, *P* < 0.05.
(TIF)

S7 Fig IL-6 expression and location in cerebral cortex of EV71-infected WT and TLR7^-/-^ mice. Neonatal WT and TLR7^-/-^ mice were intracranially injected with PBS or EV71. After 3 days EV71 incubation, brain sections from mice were subjected to immunostaining. (**A** and **B**) The cerebral cortex sections were immunostained with DAPI (Blue), IBA-1 (Green) and IL-6 (Red). Bar = 20 μm. Cells were observed using fluorescence confocal microscopy (A) and the IL-6 expression in IBA-1-positive (IBA-1^+^) cells was calculated (B). (**C** and **D**) The cerebral cortex sections were immunostained with DAPI (Blue), NeuN (Green) and IL-6 (Red). Bar = 20 μm. Cells were observed using fluorescence confocal microscopy (C) and the IL-6 expression in NeuN-positive (NeuN^+^) cells was calculated (D). Graphs show mean ± SD. ns, non-significant; *, *P* < 0.05.
(TIF)

S8 Fig The neuropathogenic effect in mice after R837 intracranial injection. There-day-old WT mice were intracranially injected with 10 μl PBS or 10 μl PBS containing 50 μg R837 per mouse, and then sacrificed on Day 1, 3 or 5 post-R837 administration. (**A**) Immunostaining of the brain’s cortex from day 3 or 5 post-R837 administration was probed with IL-6 (Red), GFAP (Green) and stained with DAPI (Blue). The presentative images were acquired using fluorescence microscopy. Bar = 20 μm. (**B**) The mice cerebral cortex sections were subjected to cl-Caspase-3 (Red), GFAP (Green) and DAPI (Blue) staining. The presentative images were captured using fluorescence microscopy. Bar = 20 μm. (**C**) The IL-6 protein level in CSF from mice was detected by ELISA. Graphs show mean ± SD. *, *P* < 0.05; **, *P* < 0.01.
(TIF)

S9 Fig The dynamic replication of EV71 in U251 cells. (**A** and **B**) U251 cells (2×10^6^) were seeded on a 6-well plate, then mock-infected or infected with EV71 (MOI = 0.5) for different periods. The total RNA was extracted from cells and EV71 RNA level was determined by qPCR. The *GAPDH* mRNA is used as an internal control (A). The total protein was extracted from cells. EV71 VP1 and 3C expression were detected by Western blotting analysis (B). (**C**) U251 cells (2×10^6^) were transfected with 2 μg plasmid transcribing siRNA specific to TLR7 (shTLR7) or its control (shGFP) and then mock-infected or infected with EV71 (MOI = 0.5) for 12 or 24 h. IL-8 secretion in supernatants of the cell cultures was analyzed by ELISA. Graphs show mean ± SD. ns, non-significant.
(TIF)

S10 Fig EV71-activated TLR7 signaling is independent of TLR8 expression. (**A**) WT or TLR7^-/-^ mice cerebral cortex sections were stained with TLR8 (Red), GFAP (Green), and DAPI (Blue). The presentative images were captured using fluorescence microscopy. Bar = 20 μm. (**B** and **C**) U251 cells (2×10^6^) were transfected with siRNA (50 nM) target to TLR8 (siRNA-TLR8) or its control (siRNA-NC) for 24 h, and then treated with EV71 (MOI = 0.5) or R837 (10 μM) for another 24 h, respectively. The cell lysates were harvested for Western blotting to examine the expression level of TLR8, TLR7, and GAPDH (B). Cell viability and LDH release of treated cells were examined using CCK8 and LDH assay, respectively (C). (**D** and **E**) U251 cells were treated with different concentrations of VXT-2337 (TLR8 agonist) (0, 1, 5 or 10 μM) for 24 h. The protein was extracted and then detected by Western blotting with targeted antibodies (D). Cell viability and LDH release of treated cells were examined using CCK8 and LDH assay (E).
(TIF)

S11 Fig The neurotoxicity effect in U251 cells after R837 treatment. (**A** and **B**) U251 cells were treated with different concentrations of R837 (0.5, 1, 5 or 10 μM) for 24 h. Cell viability (A) and LDH release (B) of treated cells were examined using CCK8 and LDH assay. (**C** and **D**) U251 cells were treated with R837 (10 μM) or EV71 (MOI = 0.5) for 24 h. The supernatants of treated cells were collected and IL-6 protein level was measured by ELISA (C). The protein was extracted and then detected by Western blotting with targeted antibodies (D). (**E** and **F**) U251 cells were seeded on 20-mm cover slips and treated with different concentrations of human IL-6 protein (20, 100, 500 pg/ml) or Etoposide (150 μM) for 24 h, and then probed with cl-Caspase-3 (Green) and DAPI (Blue) (E). Cells were observed using fluorescence confocal microscopy. Bar = 20 μm. The relative cl-Caspase-3 expression was calculated (F). Graphs show mean ± SD. *, *P* < 0.05; **, *P* < 0.01.
(TIF)

S12 Fig Histopathological characteristics in brain tissues from EV71-infected humans. (**A**) The hematoxylin-eosin (H&E) staining of cerebellum sections from human brain tissues (uninfected and EV71-infected; each group, n = 3). The presentative images were acquired using light microscopy. Bar = 100 μm. (**B**–**D**) Cerebellum sections from EV71-infected or uninfected humans were subjected to IHC staining with anti-EV71 VP1 antibody (B), anti-dsRNA antibody (C) or anti-TLR7 antibody (D). The presentative images were acquired using light microscopy. Bar = 100 μm. The relative expression of indicated was shown as a positive index and quantified with Image J software. (**E** and **F**) Human cerebellum tissue sections were fixed and stained with GFAP (Green) and IL-6 (Red) (E) or cl-Caspase-3 (Red) (F). The presentative images were acquired using fluorescence microscopy. Bar = 100 μm. The relative expression of IL-6 or cl-Caspase-3 in GFAP positive (GFAP^+^) cells was calculated with Image J software. Data are shown as mean ± SD. *, *P* < 0.05; **, *P* < 0.01; ***, *P* < 0.001.
(TIF)

S1 Table List of primers used for qPCR in this study. (DOCX)
