# Supplementary figures and images for: EV71 infection induces neurodegeneration via activating TLR7 signaling and IL-6 production
Source: PLoS Pathog. 2019 Nov 15;15(11):e1008142. doi: 10.1371/journal.ppat.1008142 (PMC6932824; doi:10.1371/journal.ppat.1008142)

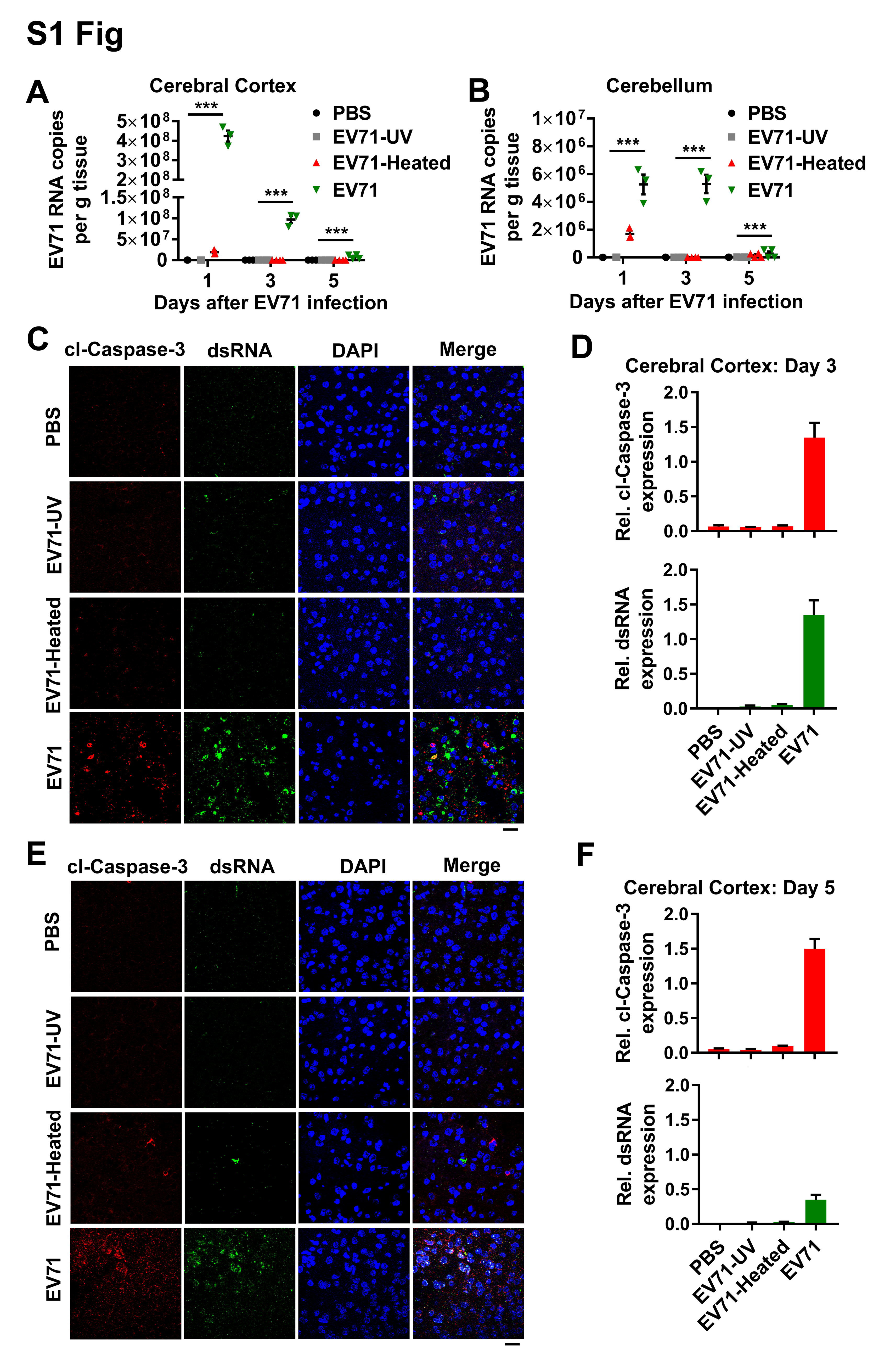

Supplement: S1 Fig — There-day-old WT mice were intracranially injected with 10 μl PBS, EV71-UV, EV71- Heated or EV71 per mouse (each group, n = 10–12) and sacrificed on day 1, 3 or 5 post-infection, respectively. (A and B) The EV71 virus RNA copies in cerebral cortex (A) or cerebellum (B) were determined by absolute quantitative PCR. Data are shown as mean ± SD. ***, P < 0.001. (C and D) The cerebral cortex sections of mice on day 3 post-infection from different groups were fixed and subjected to immunostaining with cl-Caspase-3 (Red), dsRNA (Green), and DAPI (Blue) (C). The presentative images were acquired using fluorescence microscopy. Bar = 20 μm. The relative expression of cl-Caspase-3 and dsRNA was quantified using Image J software (D). Data are shown as mean ± SD. (E and F) The cerebral cortex sections of mice on day 5 post-infection from different groups were immunostained with cl-Caspase-3 (Red), dsRNA (Green), and DAPI (Blue) (E). The presentative images were acquired using fluorescence microscopy. Bar = 20 μm. The relative expression of cl-Caspase-3 and dsRNA was quantified using Image J software (F). Data are shown as mean ± SD. (TIF) [file ppat.1008142.s001.tif]

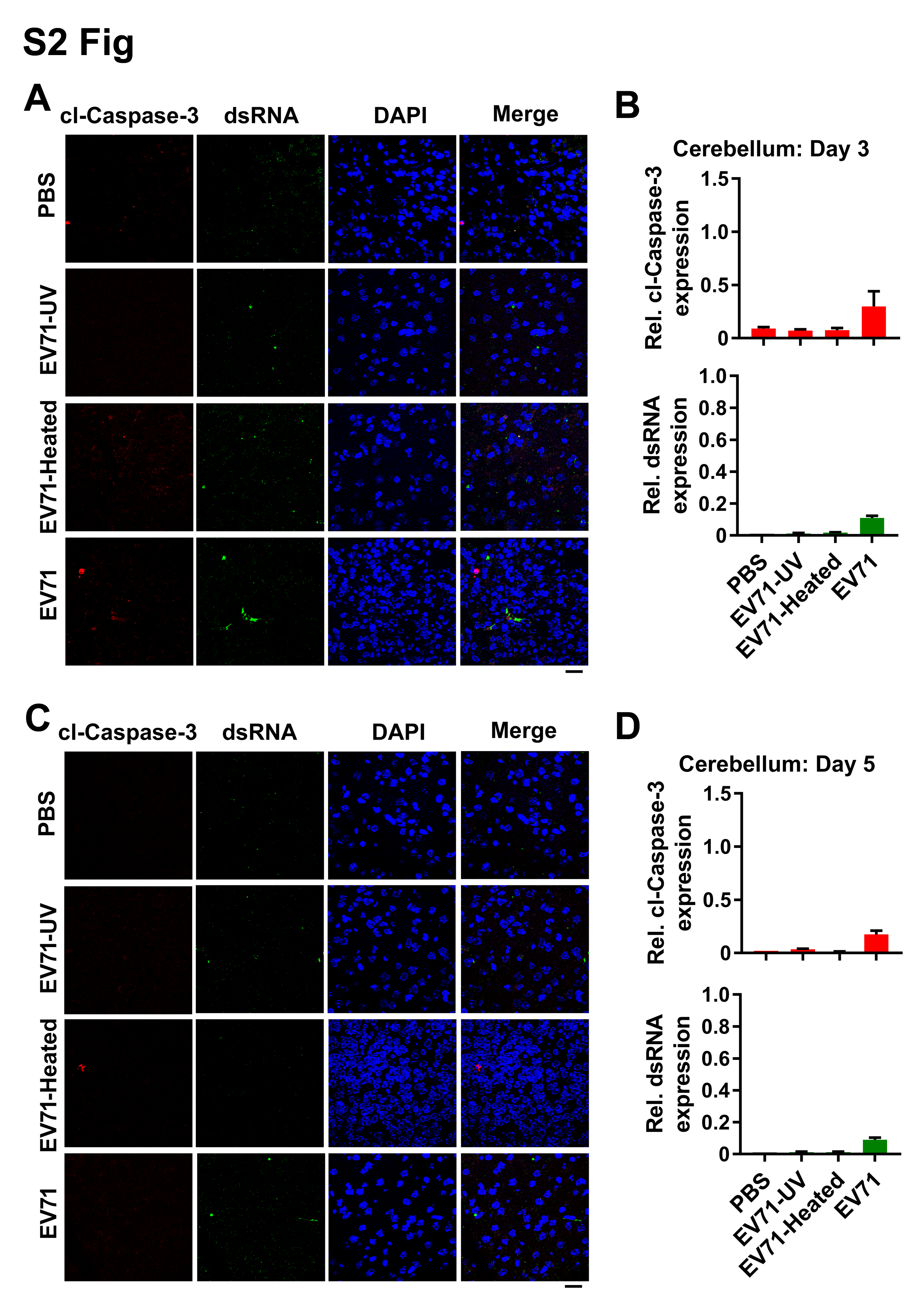

Supplement: S2 Fig — There-day-old WT mice were intracranially injected with 10 μl PBS, EV71-UV, EV71- Heated or EV71 per mouse (each group, n = 10–12) and sacrificed on day 1, 3 or 5 post-infection, respectively. (A and B) The cerebellum sections of mice on day 3 post-infection from different groups were fixed and subjected to immunostaining with cl-Caspase-3 (Red), dsRNA (Green), and DAPI (Blue) (A). The presentative images were acquired using fluorescence microscopy. Bar = 20 μm. The relative expression of cl-Caspase-3 and dsRNA was quantified using Image J software (B). Data are shown as mean ± SD. (C and D) The cerebellum sections of mice on day 5 post-infection from different groups were immunostained with cl-Caspase-3 (Red), dsRNA (Green), and DAPI (Blue) (C). The presentative images were acquired using fluorescence microscopy. Bar = 20 μm. The relative expression of cl-Caspase-3 and dsRNA was quantified using Image J software (D). Data are shown as mean ± SD. (TIF) [file ppat.1008142.s002.tif]

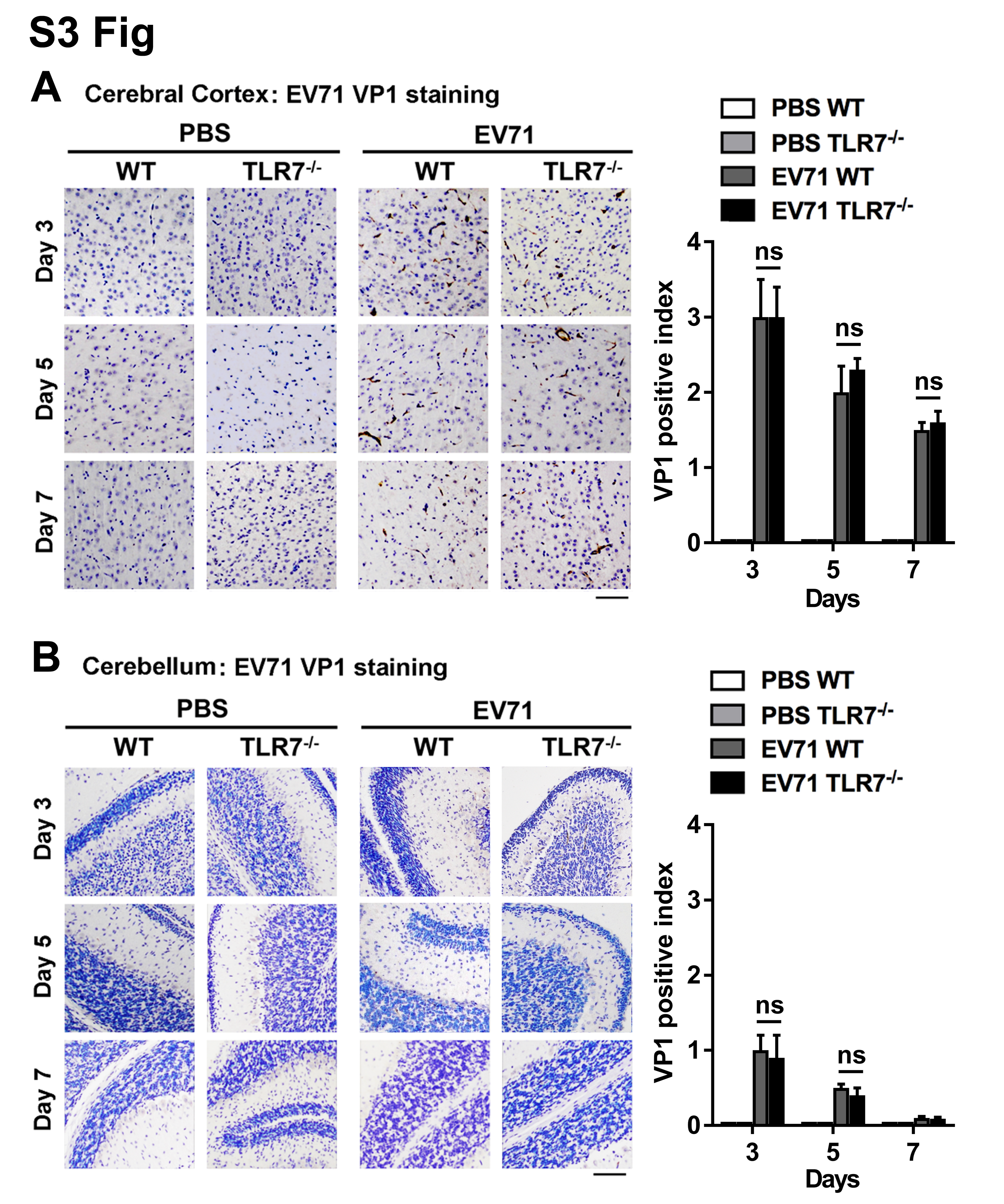

Supplement: S3 Fig — (A and B) WT mice and TLR7-/- mice mock-infected or EV71-infected were sacrificed on 2, 3, 5, and 7 days post-infection (each group, n = 3–5). The mice cerebral cortex sections (A) and cerebellum sections (B) were fixed and subjected to IHC staining with EV71 VP1 antibody (Brown), respectively. The presentative images were acquired using light microscopy. Bar = 100 μm. EV71 VP1 relative expression was shown as VP1 positive index and quantified with Image J software. Data are shown as mean ± SD. ns, non-significant. (TIF) [file ppat.1008142.s003.tif]

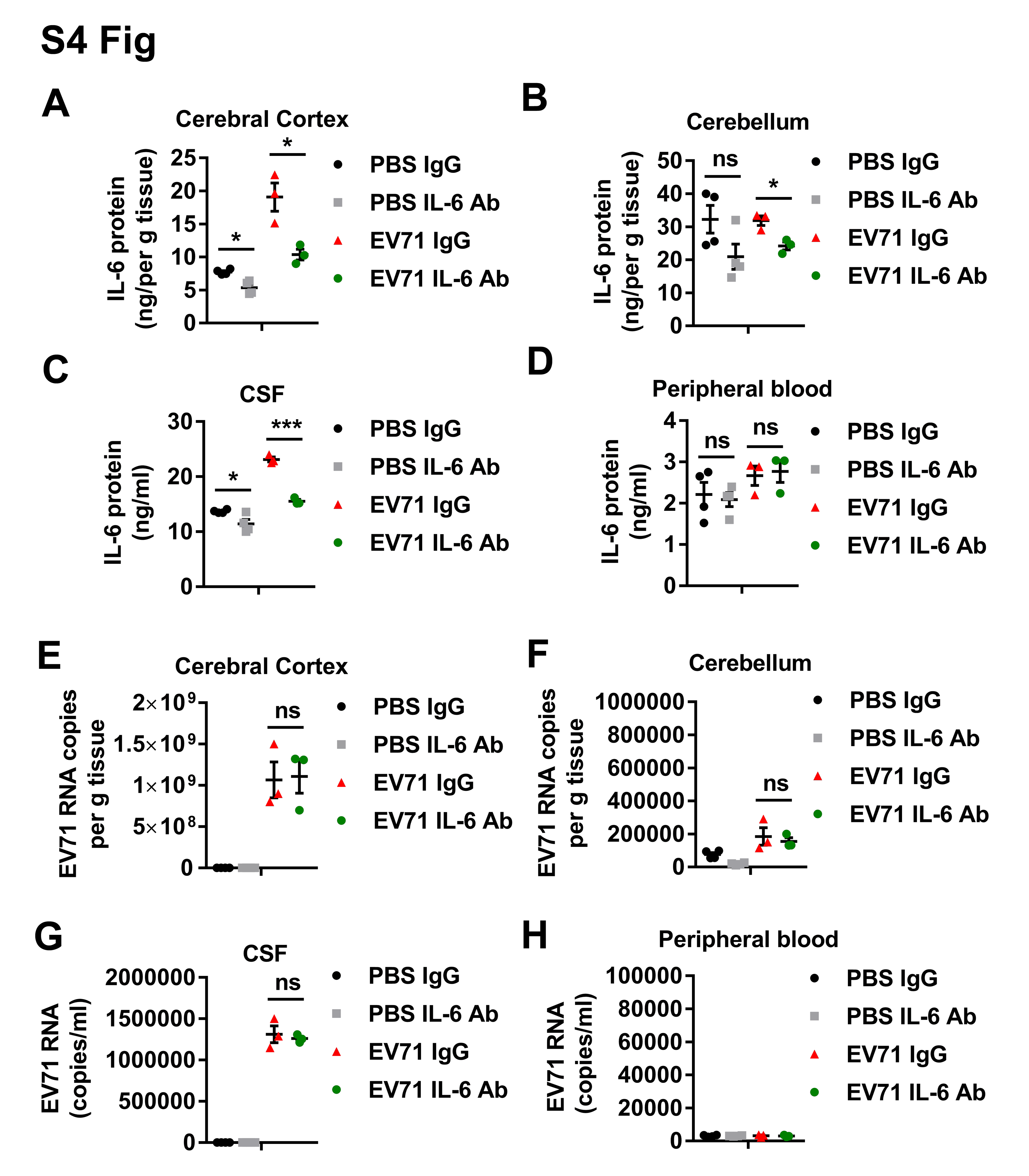

Supplement: S4 Fig — Neonatal WT mice were intracranially injected with 10 μl PBS or EV71 per mouse, and separately intracranially treated with IgG isotype or anti-IL-6 antibody. The different sections of mice on day 1 in different groups were subjected to IL-6 protein and EV71 load detection. (A and B) The proteins were extracted from individual mice cerebral cortex (A) or cerebellum (B) tissues and then the IL-6 protein level in tissues (per gram) was determined by ELISA assay. (C and D) IL-6 secretion in cerebrospinal fluid (CSF) (C) and peripheral blood (D) were determined by ELISA assay. (E-H) EV71 RNA was extracted from mice cerebral cortex (E), cerebellum (F), CSF (G) and peripheral blood (H). EV71 viral RNA copies were determined by absolute quantitative PCR. Data are shown as mean ± SD. ns, non-significant; *, P < 0.05; **, P < 0.01; ***, P < 0.001. (TIF) [file ppat.1008142.s004.tif]

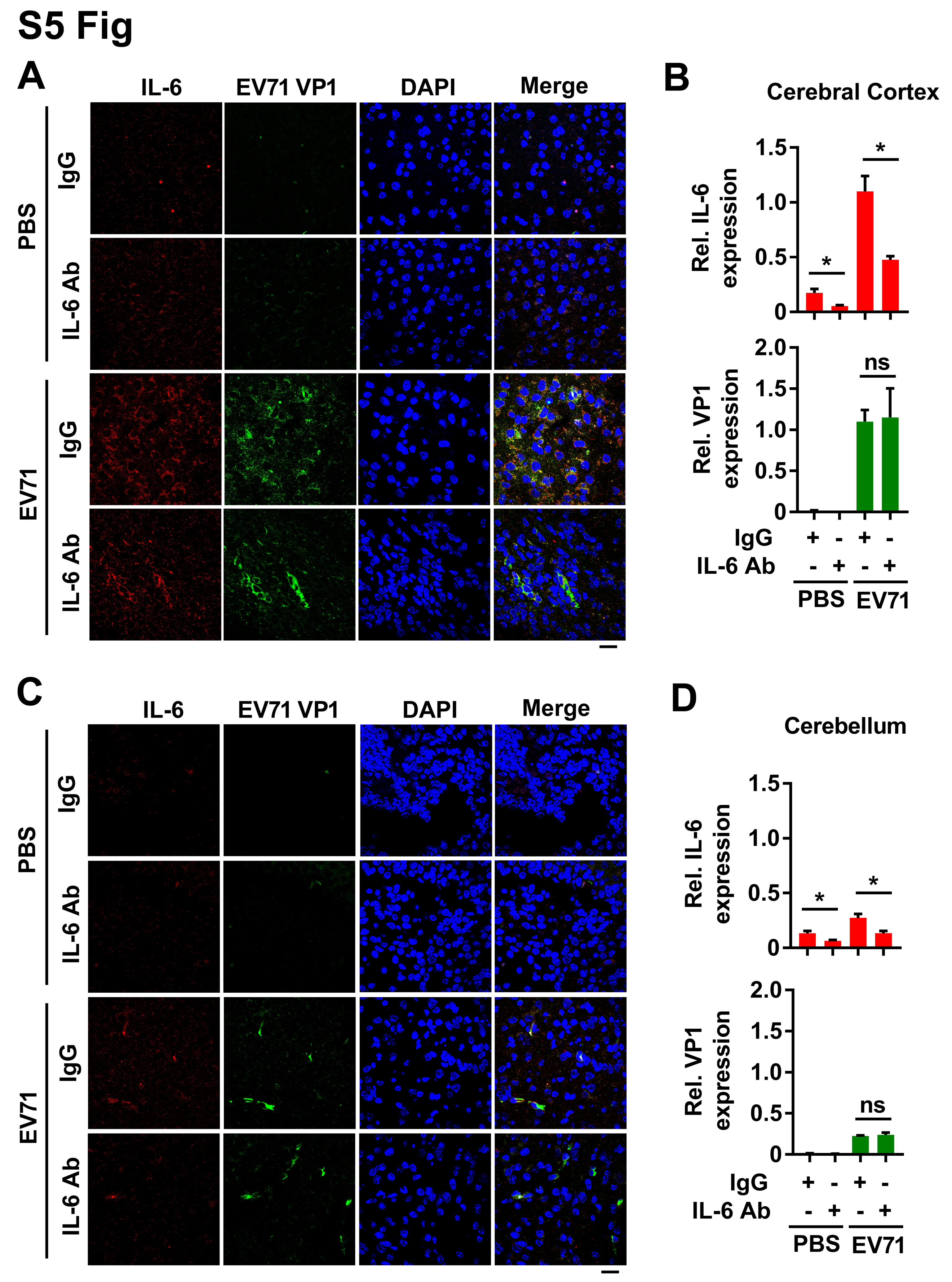

Supplement: S5 Fig — Neonatal WT mice were intracranially injected with PBS or EV71 per mouse, and separately intracranially treated with IgG isotype or anti-IL-6 antibody. The cerebral cortex and cerebellum sections of mice on day 1 in different groups were immunostained with IL-6 (Red), EV71 VP1 (Green), and DAPI (Blue). (A) The presentative images of cerebral cortex sections were acquired using fluorescence microscopy. Bar = 20 μm. (B) The relative expression of IL-6 and EV71 VP1 in cerebral cortex was quantified using Image J software. (C) The presentative images of cerebellum sections were acquired using fluorescence microscopy. Bar = 20 μm. (D) The relative expression of IL-6 and EV71 VP1 in cerebellum was quantified using Image J software. Data are shown as mean ± SD. ns, non-significant; *, P < 0.05. (TIF) [file ppat.1008142.s005.tif]

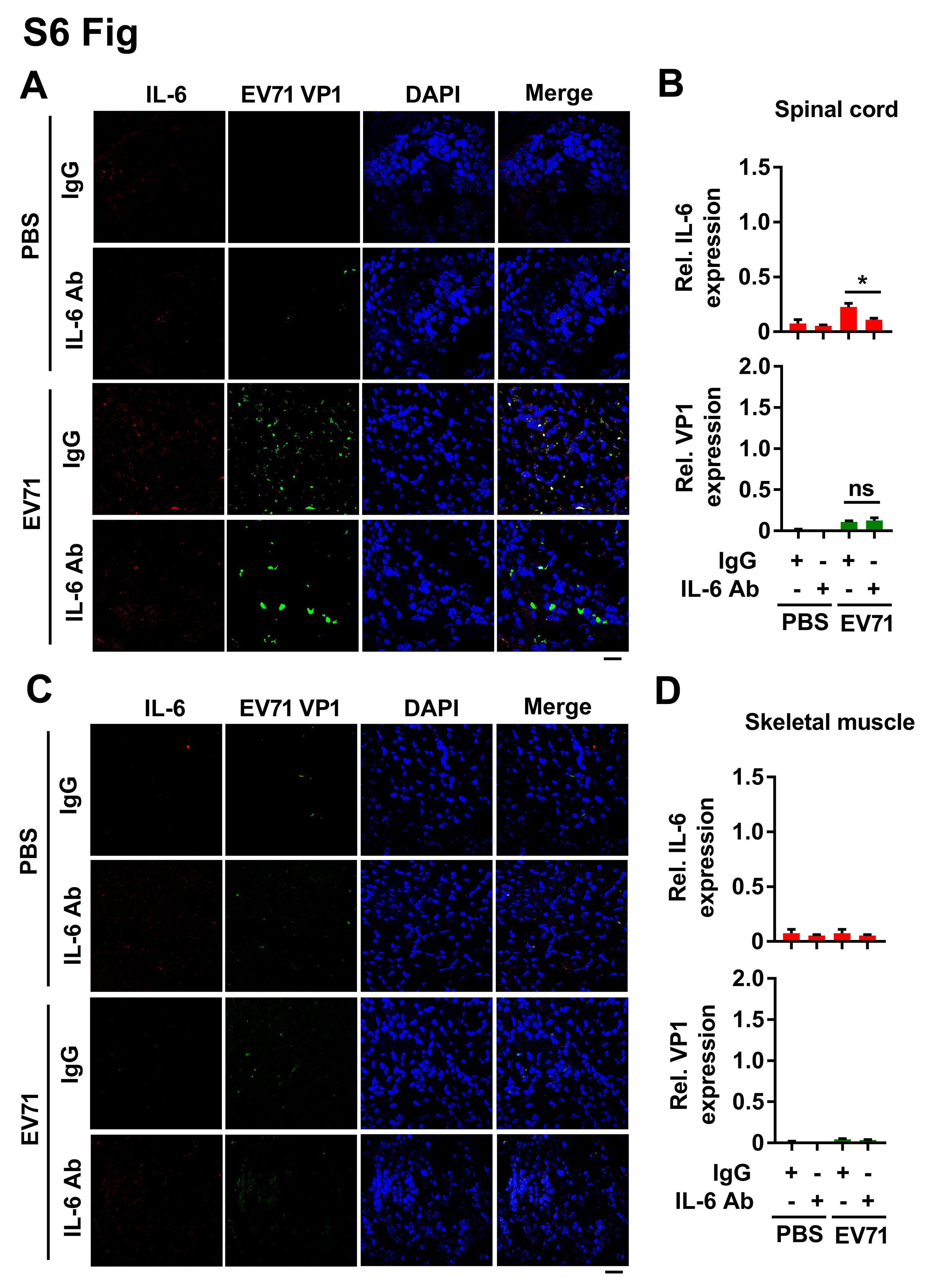

Supplement: S6 Fig — Neonatal WT mice were intracranially injected with PBS or EV71 per mouse, and separately intracranially treated with IgG isotype or anti-IL-6 antibody. The spinal cord and skeletal muscle sections of mice on day 1 in different groups were immunostained with IL-6 (Red), EV71 VP1 (Green), and DAPI (Blue). (A) The presentative images of spinal cord sections were acquired using fluorescence microscopy. Bar = 20 μm. (B) The relative expression of IL-6 and EV71 VP1 in spinal cord was quantified using Image J software. (C) The presentative images of skeletal muscle sections were acquired using fluorescence microscopy. Bar = 20 μm. (D) The relative expression of IL-6 and EV71 VP1 in skeletal muscle was quantified using Image J software. Data are shown as mean ± SD. ns, non-significant; *, P < 0.05. (TIF) [file ppat.1008142.s006.tif]

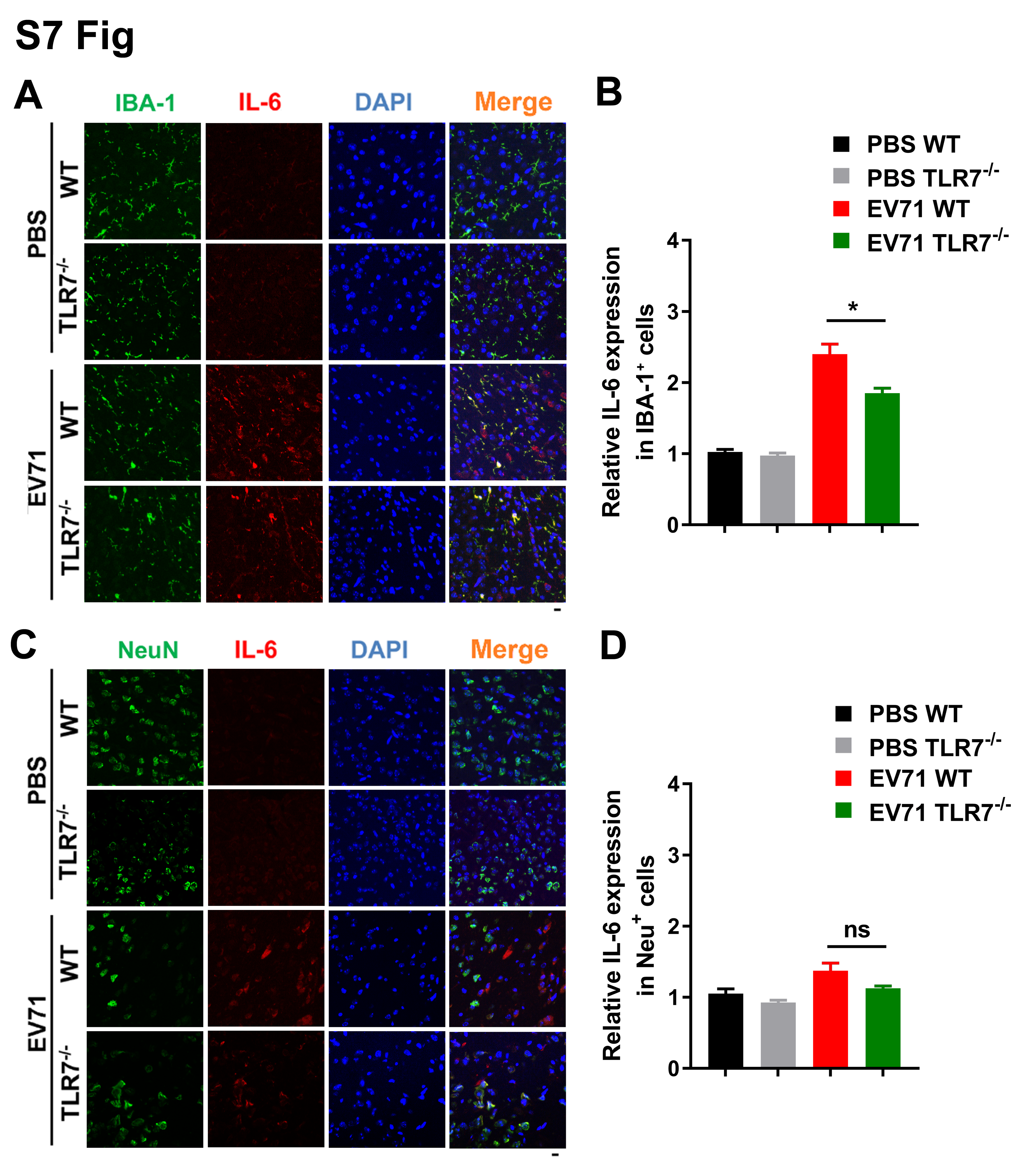

Supplement: S7 Fig — Neonatal WT and TLR7-/- mice were intracranially injected with PBS or EV71. After 3 days EV71 incubation, brain sections from mice were subjected to immunostaining. (A and B) The cerebral cortex sections were immunostained with DAPI (Blue), IBA-1 (Green) and IL-6 (Red). Bar = 20 μm. Cells were observed using fluorescence confocal microscopy (A) and the IL-6 expression in IBA-1-positive (IBA-1+) cells was calculated (B). (C and D) The cerebral cortex sections were immunostained with DAPI (Blue), NeuN (Green) and IL-6 (Red). Bar = 20 μm. Cells were observed using fluorescence confocal microscopy (C) and the IL-6 expression in NeuN-positive (NeuN+) cells was calculated (D). Graphs show mean ± SD. ns, non-significant; *, P < 0.05. (TIF) [file ppat.1008142.s007.tif]

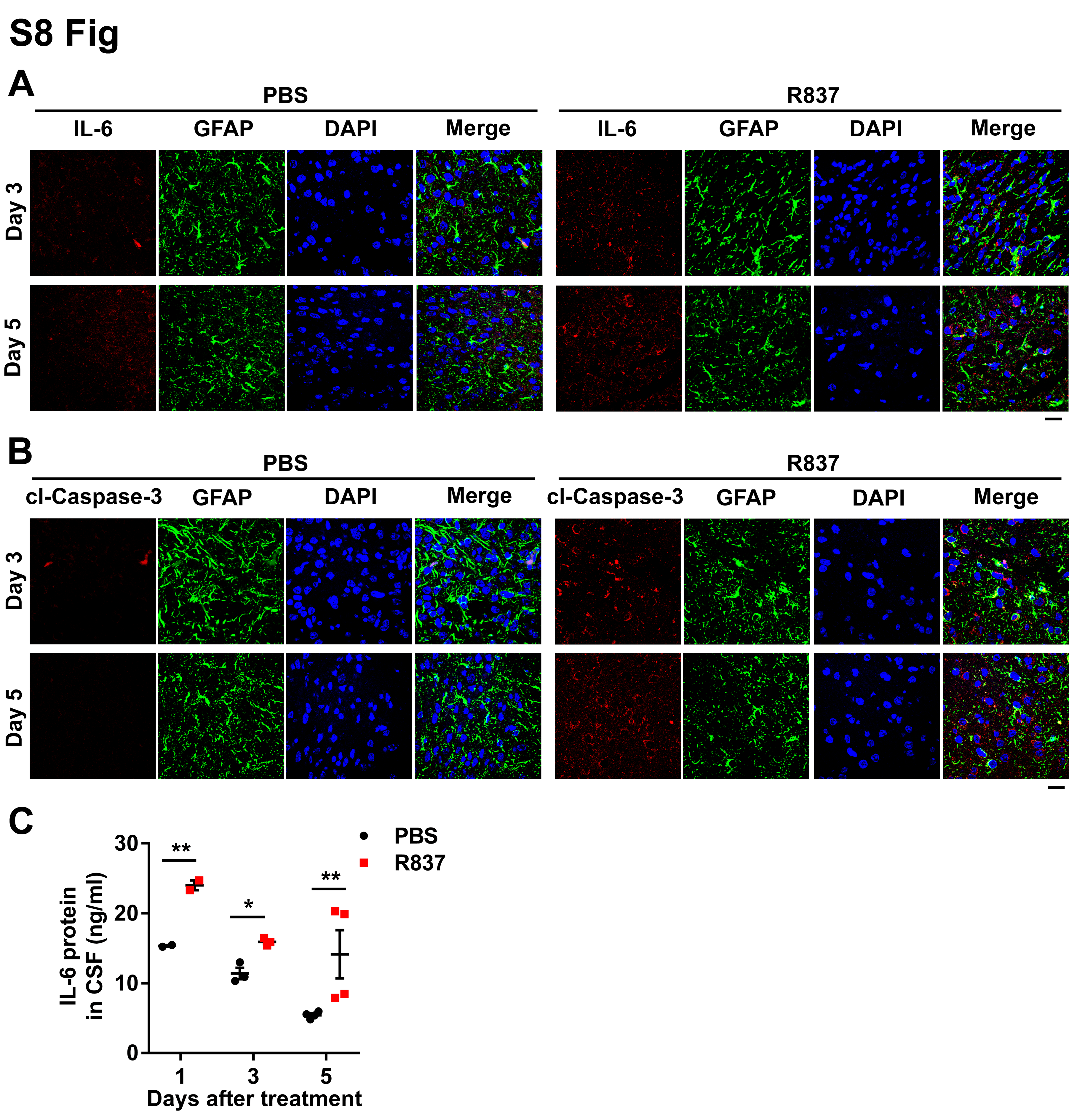

Supplement: S8 Fig — There-day-old WT mice were intracranially injected with 10 μl PBS or 10 μl PBS containing 50 μg R837 per mouse, and then sacrificed on Day 1, 3 or 5 post-R837 administration. (A) Immunostaining of the brain’s cortex from day 3 or 5 post-R837 administration was probed with IL-6 (Red), GFAP (Green) and stained with DAPI (Blue). The presentative images were acquired using fluorescence microscopy. Bar = 20 μm. (B) The mice cerebral cortex sections were subjected to cl-Caspase-3 (Red), GFAP (Green) and DAPI (Blue) staining. The presentative images were captured using fluorescence microscopy. Bar = 20 μm. (C) The IL-6 protein level in CSF from mice was detected by ELISA. Graphs show mean ± SD. *, P < 0.05; **, P < 0.01. (TIF) [file ppat.1008142.s008.tif]

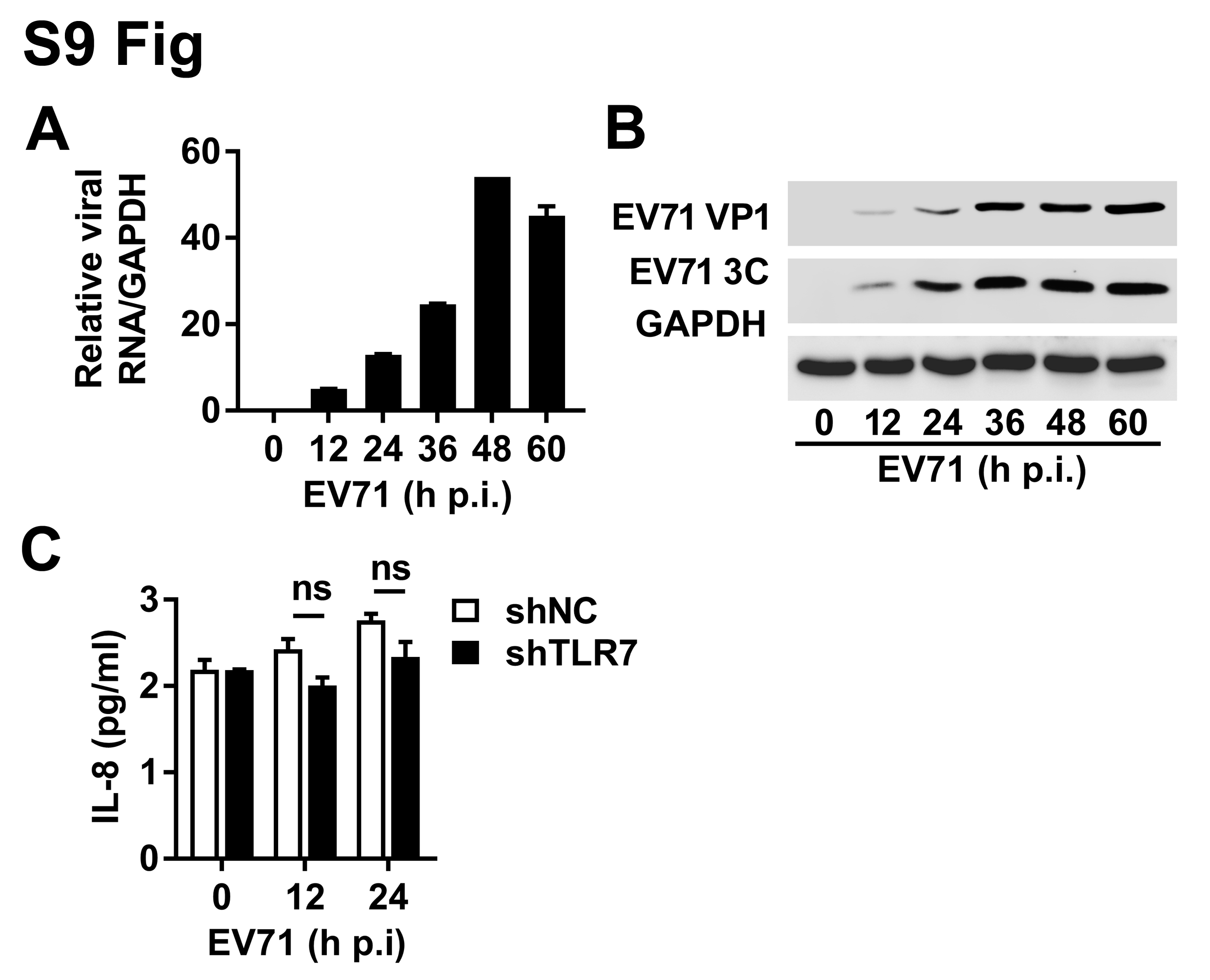

Supplement: S9 Fig — (A and B) U251 cells (2×10^6^) were seeded on a 6-well plate, then mock-infected or infected with EV71 (MOI = 0.5) for different periods. The total RNA was extracted from cells and EV71 RNA level was determined by qPCR. The GAPDH mRNA is used as an internal control (A). The total protein was extracted from cells. EV71 VP1 and 3C expression were detected by Western blotting analysis (B). (C) U251 cells (2×10^6^) were transfected with 2 μg plasmid transcribing siRNA specific to TLR7 (shTLR7) or its control (shGFP) and then mock-infected or infected with EV71 (MOI = 0.5) for 12 or 24 h. IL-8 secretion in supernatants of the cell cultures was analyzed by ELISA. Graphs show mean ± SD. ns, non-significant. (TIF) [file ppat.1008142.s009.tif]

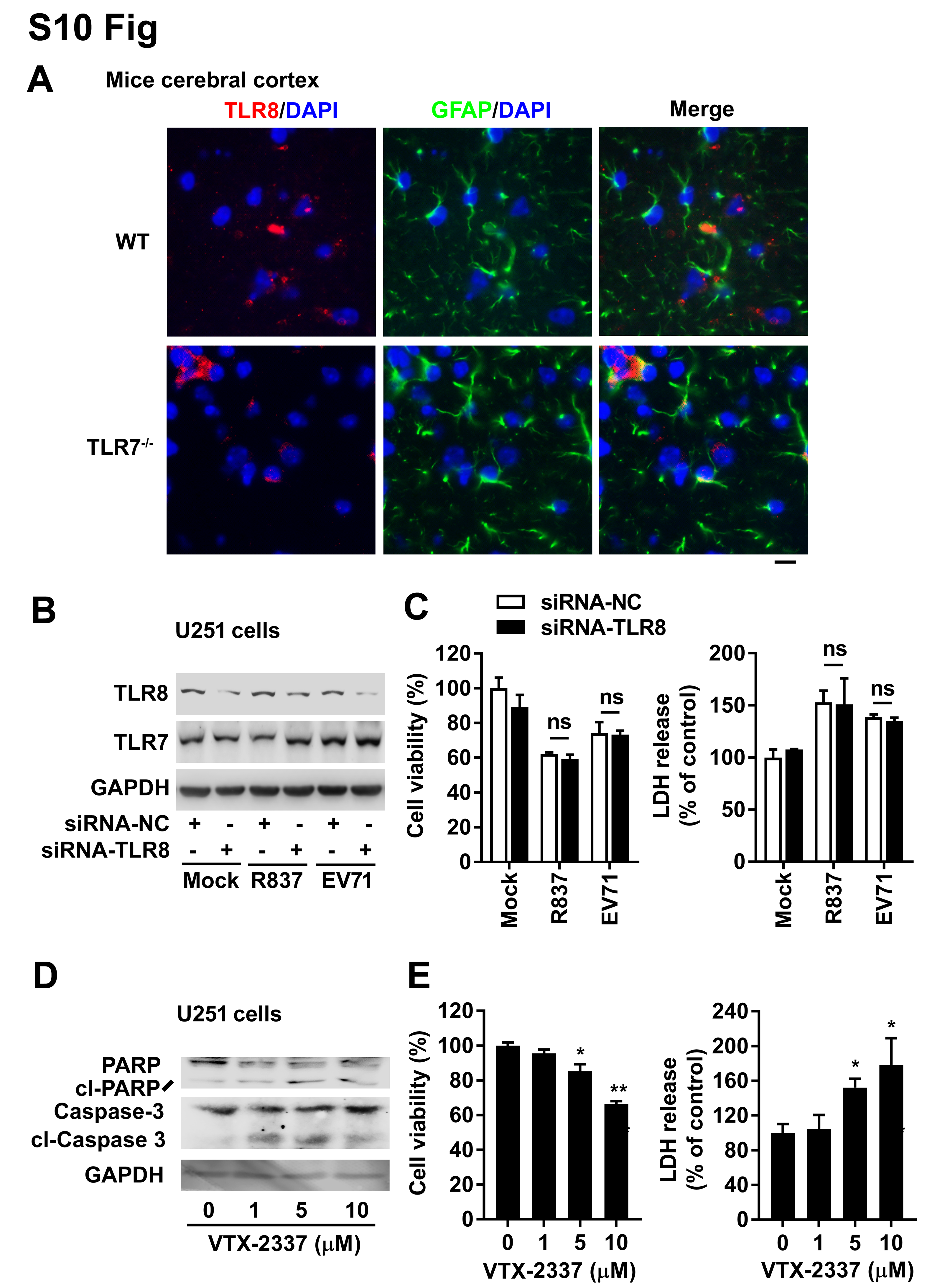

Supplement: S10 Fig — (A) WT or TLR7-/- mice cerebral cortex sections were stained with TLR8 (Red), GFAP (Green), and DAPI (Blue). The presentative images were captured using fluorescence microscopy. Bar = 20 μm. (B and C) U251 cells (2×10^6^) were transfected with siRNA (50 nM) target to TLR8 (siRNA-TLR8) or its control (siRNA-NC) for 24 h, and then treated with EV71 (MOI = 0.5) or R837 (10 μM) for another 24 h, respectively. The cell lysates were harvested for Western blotting to examine the expression level of TLR8, TLR7, and GAPDH (B). Cell viability and LDH release of treated cells were examined using CCK8 and LDH assay, respectively (C). (D and E) U251 cells were treated with different concentrations of VXT-2337 (TLR8 agonist) (0, 1, 5 or 10 μM) for 24 h. The protein was extracted and then detected by Western blotting with targeted antibodies (D). Cell viability and LDH release of treated cells were examined using CCK8 and LDH assay (E). (TIF) [file ppat.1008142.s010.tif]

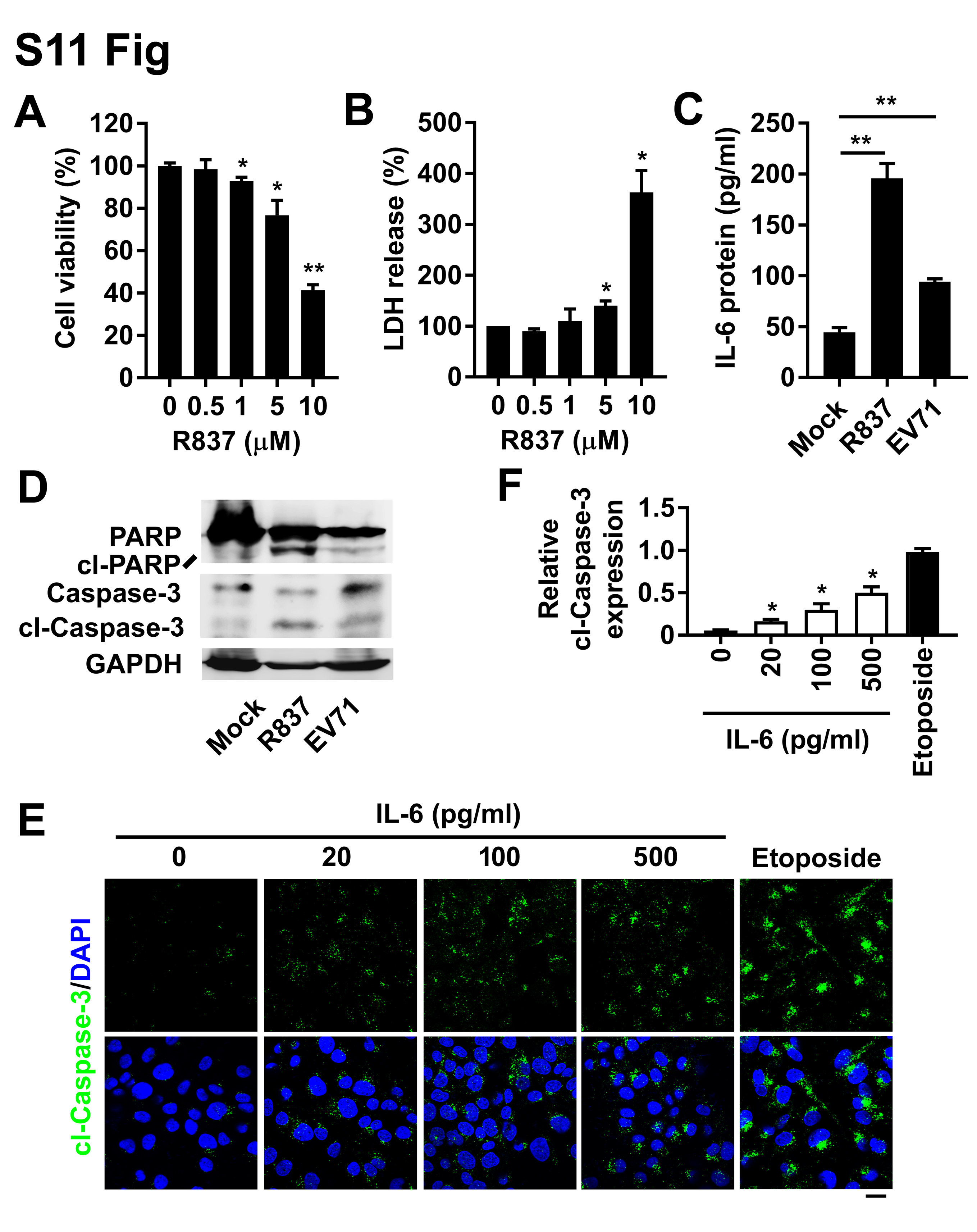

Supplement: S11 Fig — (A and B) U251 cells were treated with different concentrations of R837 (0.5, 1, 5 or 10 μM) for 24 h. Cell viability (A) and LDH release (B) of treated cells were examined using CCK8 and LDH assay. (C and D) U251 cells were treated with R837 (10 μM) or EV71 (MOI = 0.5) for 24 h. The supernatants of treated cells were collected and IL-6 protein level was measured by ELISA (C). The protein was extracted and then detected by Western blotting with targeted antibodies (D). (E and F) U251 cells were seeded on 20-mm cover slips and treated with different concentrations of human IL-6 protein (20, 100, 500 pg/ml) or Etoposide (150 μM) for 24 h, and then probed with cl-Caspase-3 (Green) and DAPI (Blue) (E). Cells were observed using fluorescence confocal microscopy. Bar = 20 μm. The relative cl-Caspase-3 expression was calculated (F). Graphs show mean ± SD. *, P < 0.05; **, P < 0.01. (TIF) [file ppat.1008142.s011.tif]

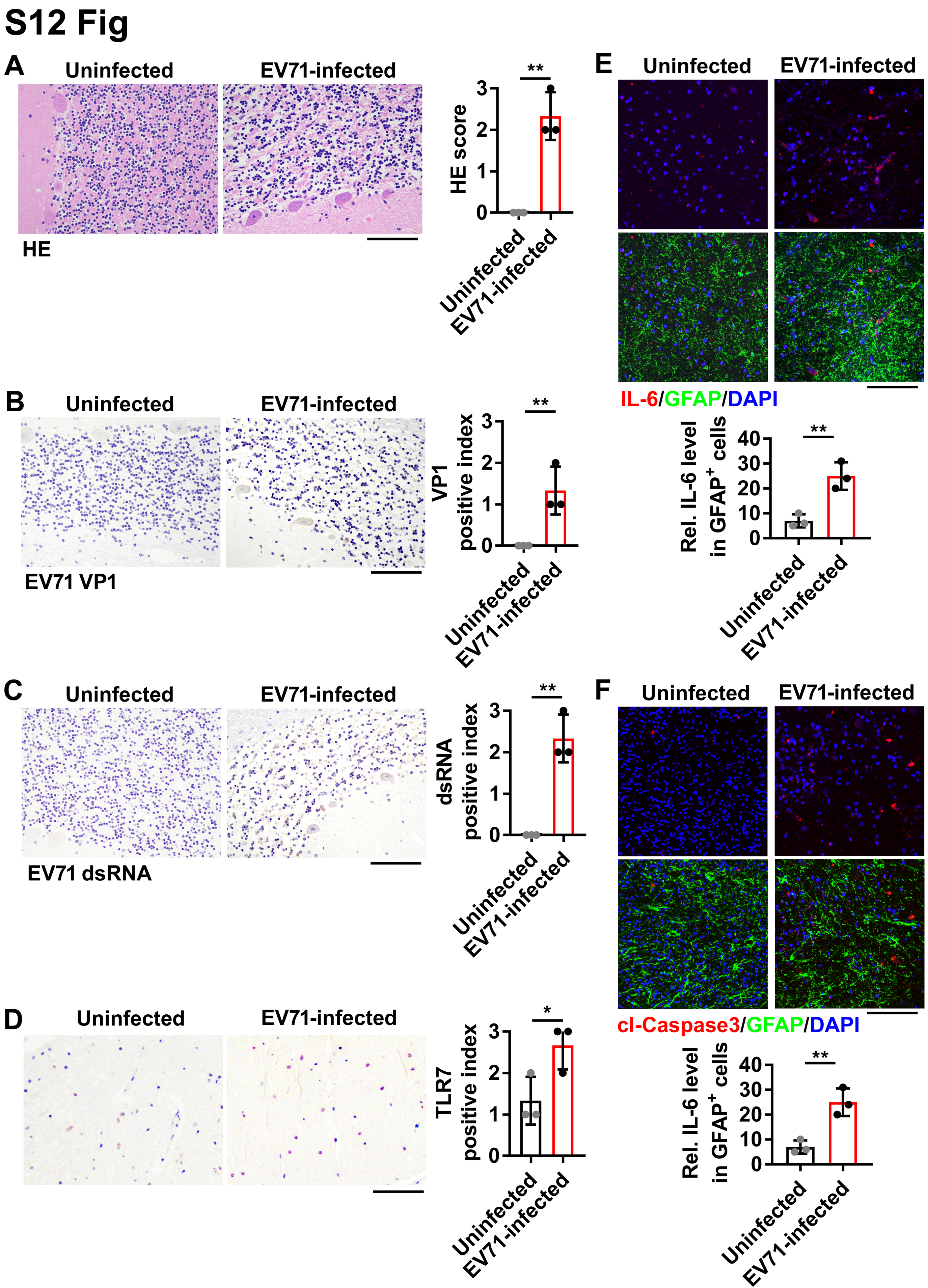

Supplement: S12 Fig — (A) The hematoxylin-eosin (H&E) staining of cerebellum sections from human brain tissues (uninfected and EV71-infected; each group, n = 3). The presentative images were acquired using light microscopy. Bar = 100 μm. (B–D) Cerebellum sections from EV71-infected or uninfected humans were subjected to IHC staining with anti-EV71 VP1 antibody (B), anti-dsRNA antibody (C) or anti-TLR7 antibody (D). The presentative images were acquired using light microscopy. Bar = 100 μm. The relative expression of indicated was shown as a positive index and quantified with Image J software. (E and F) Human cerebellum tissue sections were fixed and stained with GFAP (Green) and IL-6 (Red) (E) or cl-Caspase-3 (Red) (F). The presentative images were acquired using fluorescence microscopy. Bar = 100 μm. The relative expression of IL-6 or cl-Caspase-3 in GFAP positive (GFAP+) cells was calculated with Image J software. Data are shown as mean ± SD. *, P < 0.05; **, P < 0.01; ***, P < 0.001. (TIF) [file ppat.1008142.s012.tif]
