# Supplementary material for: EV71 infection induces neurodegeneration via activating TLR7 signaling and IL-6 production
Source: PLoS Pathog. 2019 Nov 15;15(11):e1008142. doi: 10.1371/journal.ppat.1008142 (PMC6932824; doi:10.1371/journal.ppat.1008142)
Supplement: S1 Table — (DOCX) [file ppat.1008142.s013.docx]

**S1 Table. List of primers used for qPCR in this study.**

| Primer title | Orientation |
| --- | --- |
| qmTLR7 F | 5’-GTTCTATGGAGAGCCGGTGATA-3’ |
| qmTLR7 R | 5’-ATTCTTTAGATTTGGCGGCATA-3’ |
| qmIL-1β F | 5’-AGCTTCAGGCAGGCAGTATC-3’ |
| qmIL-1β R | 5’-CGTCACACACCAGCAGGTTA-3’ |
| qmIL-6 F | 5’-AGACAAAGCCAGAGTCCTTCAGAGA-3’ |
| qmIL-6 R | 5’-GCCACTCCTTCTGTGACTCCAGC-3’ |
| qmCxcl-1 F | 5’-CTTGAAGGTGTTGCCCTCAG-3’ |
| qmCxcl-1 R | 5’-TGGGGACACCTTTTAGCATC-3’ |
| qmTNFα F | 5’-ACGTGGAACTGGCAGAAGAG-3’ |
| qmTNFα R | 5’-CTCCTCCACTTGGTGGTTTG-3’ |
| qmGAPDH F | 5’-ATGTTTGTGATGGGTGTGAA-3’ |
| qmGAPDH R | 5’-ATGCCAAAGTTGTCATGGAT-3’ |
| EV71 VP1 F | 5’-GAGTTCCATAGGTGACAGC-3’ |
| EV71 VP1 R | 5’-CTGTGCGAATTAAGGACAG-3’ |
| qmPUMA F | 5’-ACGAGCGGCGGAGACAAGAAG-3’ |
| qmPUMA R | 5’-CAGAAGGCGGAGGGGGTCCT-3’ |
| qmBIM F | 5’-CGGCGTCTGCGTGGTGATTC-3’ |
| qmBIM R | 5’-GCACAGGCAGTTCCCGACCC-3’ |
| qmBID F | 5’-CGCTCCTTCAACCAAGGAAG-3’ |
| qmBID R | 5’-GTGGTCCATCTCATCGCCTA-3’ |
| qmBAD F | 5’-GCGATGAGTTTGAGGGTTCC-3’ |
| qmBAD R | 5’-GGAGCAGAAGATCACTGGGA-3’ |
| qmNOXA F | 5’-GAGTTCGCAGCTCAACTCAG-3’ |
| qmNOXA R | 5’-GCACACTCGTCCTTCAAGTC-3’ |
| qmBAX F | 5’-CGTGGTTGCCCTCTTCTACT-3’ |
| qmBAX R | 5’-TGATCAGCTCGGGCACTTTA-3’ |
| qmBAK F | 5’-AGATGGATCGCACAGAGAGG-3’ |
| qmBAK R | 5’-AATTGGCCCAACAGAACCAC-3’ |
| qmCytC F | 5’-GAGGCAAGCATAAGACTGG-3’ |
| qmCytC R | 5’-TACTCCATCAGGGTATCCT-3’ |
| qmSMAC F | 5’-TGTTCAGGTACAGACAGCGT-3’ |
| qmSMAC R | 5’-AGCCCTCCTCATCAATGCTT-3’ |
| qmXIAP F | 5’- GCAATGTTTCAGTTGTCAT-3’ |
| qmXIAP R | 5’-TCGGGTATATGGTGTCTGAAA-3’ |
| qmAPAF1 F | 5’-TCAGATGGCCAGCTTCTTCA-3’ |
| qmAPAF1 R | 5’-GTGGCAACATTCCACCACTT-3’ |
| qmBCL2 F | 5’- GTGTTCCATGCACCAAGTCCA-3’ |
| qmBCL2 R | 5’-AGGTACAGGCATTGCCGCATA-3’ |
| qmBCL-XL F | 5’-CGTGGAAAGCGTAGACAAGG-3’ |
| qmBCL-XL R | 5’-GCTGCATTGTTCCCGTAGAG-3’ |
| qmBCL-W F | 5’-ACTGGGGCCGTCTTGTGGCA-3’ |
| qmBCL-W R | 5’-GAGCTGTGAACTCCGCCCAGC-3’ |
| qmCaspase3 F | 5’ -TGGTGATGAAGGGGTCATTTATG-3’ |
| qmCaspase3 R | 5’ -TTCGGCTTTCCAGTCAGACTC-3’ |
| qmCaspase9 F | 5’-AGTTCCCGGGTGCTGTCTA-3’ |
| qmCaspase9 R | 5’-GCCATGGTCTTTCTGCTCA-3’ |
| qmCaspase7 F | 5’-AGAGGACTTCGGTTCCCTTG-3’ |
| qmCaspase7 R | 5’-TGGTTTCCATCTCAGGCAGT-3’ |
| qmMCL1 F | 5’-TTCTTTCGGTGCCTTTGTGG-3’ |
| qmMCL1 R | 5’-AAACCCATCCCAGCCTCTTT-3’ |
| qhTLR7 | 5’-TTTACCTGGATGGAAACCAGCTA-3’ |
| qhTLR7 | 5’-TCAAGGCCTGAGAAGCTGTAAGCTA-3’ |
| qhIL-1β F | 5’-AACCTGCTGGTGTGTGACGTTC-3’ |
| qhIL-1β R | 5’-AGCACGAGGCTTTTTTGTTGT-3’ |
| qhIL-6 F | 5’-GTACATCCTCGACGGCATCTCA-3’ |
| qhIL-6 R | 5’-GCACAGCTCTGGCTTGTTCCTC-3’ |
| qhIL-8 F | 5’-AAGGAACCATCTCACTGTGTGTAAAC-3’ |
| qhIL-8 R | 5’-TTAGCACTCCTTGGCAAAACTG-3’ |
| qhTNFα F | 5’-TCTCGAACCCCGAGTGACA-3’ |
| qhTNFα R | 5’-GGCCGGCGGTTCA-3’ |
| qhGAPDH F | 5’-AAGGCTGTGGGCAAGG-3’ |
| qhGAPDH R | 5’-TGGAGGAGTGGGTGTCG-3’ |
| qhPUMA F | 5’-GACCTCAACGCACAGTACGAG-3’ |
| qhPUMA R | 5’-CCTAATTGGGCTCCATCTCG-3’ |
| qhBIM F | 5’-AGTTGCGGCGTATTGGAG-3’ |
| qhBIM R | 5’-ATTCGTGGGTGGTCTTCG-3’ |
| qhBID F | 5’-CGTCCTTGCTCCGTGATG-3’ |
| qhBID R | 5’-ATGCCAGGGCTCCGTCTA-3’ |
| qhBAD F | 5’-CGGAGGATGAGTGACGAGTTT-3’ |
| qhBAD R | 5’-CGGGATGTGGAGCGAAGGT-3’ |
| qhNOXA F | 5’-GACACCCGATCCCAGCAT-3’ |
| qhNOXA R | 5’-CTCGACTTCCAGCTCTGC-3’ |
| qhBAX F | 5’-AGGGTGGTTGGGTGAGACT-3’ |
| qhBAX R | 5’-GGGAGGTCAGCAGGGTAGAT-3’ |
| qhBAK F | 5’-GAGCAGGTAGCCCAGGACA-3’ |
| qhBAK R | 5’-TAGCGTCGGTTGATGTCG-3’ |
| qhSMAC F | 5’-TTGGCGGTCGTGCCTTAT-3’ |
| qhSMAC R | 5’-TTCATCTTCCTCCTCTGAAT-3’ |
| qhAPAF1 F | 5’-TAGTGAAGTGTTGTTCGTGGTC-3’ |
| qhAPAF1 R | 5’-TGGATGGTGCTGTGATGG-3’ |
| qhCaspase3 F | 5’-GCTATTGTGAGGCGGTTGT-3’ |
| qhCaspase3 R | 5’-AGCAGGGCTCGCTAACTC-3’ |
| qhMCL1 F | 5’-AAGGATGGGTTTGTGGAG-3’ |
| qhMCL1 R | 5’-CTAGGTTGCTAGGGTGCA-3’ |

The sequences of primer involved in this study are listed. F, forward; R, reverse; qh (qm), the primers for human (mouse) genes used in quantitative real-time PCR.
